# The Role of the FOXO1/β_2_-AR/p-NF-κB p65 Pathway in the Development of Endometrial Stromal Cells in Pregnant Mice under Restraint Stress

**DOI:** 10.3390/ijms22031478

**Published:** 2021-02-02

**Authors:** Jiayin Lu, Yaoxing Chen, Zixu Wang, Jing Cao, Yulan Dong

**Affiliations:** 1 Laboratory of Neurobiology, College of Veterinary Medicine, China Agricultural University, Beijing 100193, China; lujiayin0324@cau.edu.cn (J.L.); yxchen@cau.edu.cn (Y.C.); zxwang@cau.edu.cn (Z.W.); caojing@cau.edu.cn (J.C.); 2 Key Laboratory of Precision Nutrition and Food Quality, Ministry of Education, China Agricultural University, Beijing 100193, China

**Keywords:** restraint stress, pregnancy, FOXO, β_2_-AR, p-NF-κB p65

## Abstract

Restraint stress causes various maternal diseases during pregnancy. β_2_-Adrenergic receptor (β_2_-AR) and Forkhead transcription factor class O 1 (FOXO1) are critical factors not only in stress, but also in reproduction. However, the role of FOXO1 in restraint stress, causing changes in the β_2_-AR pathway in pregnant mice, has been unclear. The aim of this research was to investigate the β_2_-AR pathway of restraint stress and its impact on the oxidative stress of the maternal uterus. In the study, maternal mice were treated with restraint stress by being restrained in a transparent and ventilated device before sacrifice on Pregnancy Day 5 (P5), Pregnancy Day 10 (P10), Pregnancy Day 15 (P15), and Pregnancy Day 20 (P20) as well as on Non-Pregnancy Day 5 (NP5). Restraint stress augmented blood corticosterone (CORT), norepinephrine (NE), and blood glucose levels, while oestradiol (E2) levels decreased. Moreover, restraint stress increased the mRNA levels of the FOXO family, β_2_-AR, and even the protein levels of FOXO1 and β_2_-AR in the uterus and ovaries. Furthermore, restraint stress increased uterine oxidative stress level. In vitro, the protein levels of FOXO1 were also obviously increased when β_2_-AR was activated in endometrial stromal cells (ESCs). In addition, phosphorylated-nuclear factor kappa-B p65 (p-NF-κB p65) and its target genes decreased significantly when FOXO1 was inhibited. Overall, it can be said that the β_2_-AR/FOXO1/p-NF-κB p65 pathway was activated when pregnant mice were under restraint stress. This study provides a scientific basis for the origin of psychological stress in pregnant women.

## 1. Introduction

Some researchers have found that psychological stress has a negative effect on pregnancy development, mainly in the study of future generations, including delayed foetal maturation [1], loss of foetal immune function [2], and offspring neuropsychiatric risk [3]. Most scholars have used the model of restraint stress to simulate psychological stress [4]. In the uterus, Liu et al. found that the balance of oxidative and antioxidative stress and the balance of apoptosis and proliferation in the uterus were altered under restraint stress [5,6]. The mechanism of this dysregulation might be due to the involvement of the nervous and endocrine systems. Gonadotropin-releasing hormone (GnRH) is inhibited by corticotropin-releasing hormone (CRH), and CRH-induced proopiomelanocortin peptides suppress ovarian oestradiol and progesterone as well as pituitary luteinizing hormone (LH) secretion [7].

Exogenous stress activates sympathetic nerve endings to release catecholamine-type transmitters, including norepinephrine (NE), which acts by binding to adrenergic receptors located on target organs. Both NE and corticosterone (CORT) are marker of stress, and they increase during stress response [8]. It has been reported that β_2_-AR existed in the uterus of mice four days after pregnancy. Meanwhile, the β_2_-AR signalling pathway is activated after restraint stress, resulting in an asymmetric distribution of intrauterine embryos [9]. During early pregnancy and placenta formation, local reactive oxygen species (ROS) levels in the uterus are high in order to induce oxidative stress, while the content of the antioxidant substance Total Superoxide Dismutase (T-SOD) increases, maintaining the local oxidative–antioxidant balance of the uterus [10]. However, excessive oxidative stress is considered to be responsible for the initiation or development of pathological processes affecting female reproductive activities [11,12,13], such as embryonic resorption, recurrent pregnancy loss, intrauterine growth restriction (IUGR), preeclampsia, and foetal death [13,14]. It was reported that oxidative stress may affect the pregnancy process by inducing the signalling pathway of NF-κB [15]. As is known, NF-κB can promote inflammation [16]. Meanwhile, changes in NF-κB caused by oxidative stress may also suggest an inflammatory response in the uterus [5]. However, the relationship between NF-κB and β_2_-AR is unclear. Therefore, whether NF-κB is involved in the β_2_-AR signal pathway remains to be studied.

The Forkhead transcription factor class O (FOXO) gene encodes a series of transcription factors that regulate oxidative stress, DNA repair, and apoptosis in cells in order to maintain homeostasis [17,18,19]. In addition, the important role of FOXO in the reproductive process is mainly in its ability to regulate decidualization, under the influence of progesterone content, and to regulate cell apoptosis and differentiation [20]. A study showed that the β_2_-AR agonist induced the expression of FOXO1 in mouse skeletal muscle [21]. In view of the role of FOXO in oxidative stress and decidualization, the role of FOXO in the activation of β_2_-AR should also be taken seriously.

In this study, a model of restraint stress was established to study various changes throughout pregnancy, including the detection of hormones in plasma, changes in the uterine antioxidant capacity, and the detection of membrane receptor and intracellular signalling molecules. Finally, the FOXO1-mediated β_2_-AR/NF-κB signalling pathway was examined by a cell assay. We focused on the effect of restraint stress in pregnant mothers throughout pregnancy and conducted preliminary studies of signalling pathways.

## 2. Results

### 2.1. Effect of Restraint Stress on Body Weight in Pregnant Mice

**Figure 1:**
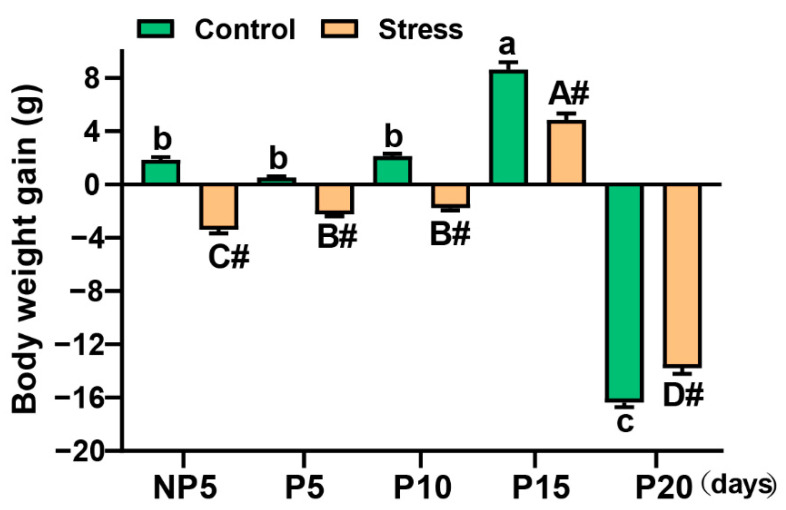
Changes in body weight after restraint stress. Restraint stress caused a significant decrease in the weight gain of pregnant mothers. The data are expressed as the mean ± standard error of mean (SEM). # was used to denote extremely significant difference between stress and corresponding control groups (*p* < 0.01). Uppercase letters in the column (A, B, C, D) represent the differences between different ages in the stress group (*p* < 0.05). A (in the P15 group) represents the biggest value in the stress. Lowercase letters in the column (a, b, c) represent the differences between different ages in the control group (*p* < 0.05). a (in the P15) represents the biggest value in the control. Treatment groups are significantly different from each other if no labelling letter (a to e or A to E) is shared between groups. The X axis represents the days of pregnancy, NP5: Non-Pregnancy Day 5, P5: Pregnancy Day 5, P10: Pregnancy Day 10, P15: Pregnancy Day 15, P20: Pregnancy Day 20. Every group has 10 mice.

Body weight was measured before (1st day) and after stress (5th day). The body weight gain (BWG) was calculated. S_BWG_ = S5^th^-S1^st^ (Stress group), C_BWG_ = C5^th^-C1^st^ (Control group). Body weight gains were affected by pregnancy age (*p* < 0.001) and restraint stress (*p* < 0.001). In this study, the body weight gain was reduced significantly after restraint stress in different ages compared with control groups (independent sample *t* test, NP5: 281.07%, *p* < 0.001; P5: 517.86%, *p* < 0.001; P10: 182.81%, *p* < 0.001; P15: 43.74%, *p* = 0.004) (Figure 1). However, on the 20th day, the mother delivered the pups, which led to the rapid decline of weight at P20. Besides, in our previous study, the number of foetuses were fewer under restraint stress than control mice [5]. So, the change of BWG in the stress group was fewer than in the control group (independent sample *t* test, P20: 15.60%, *p* = 0.006) (Figure 1) at P20 (for the specific value, see Table S1 of the Supplementary Material). Overall, restraint stress led to loss in body weight.

### 2.2. Effect of Restraint Stress on Organ Index in Pregnant Mice

**Figure 2:**
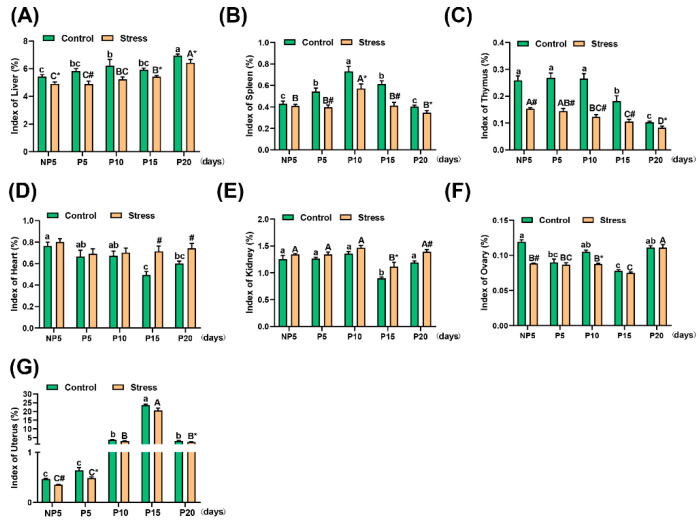
Effect of restraint stress on organ index in pregnant mice. The liver, spleen, kidney, heart, ovary, uterus, and thymus were weighed. The organ index was expressed as the ratio of the weight of the organ to the body weight. The result showed that restraint stress caused a significant decrease in the organ index of the liver (**A**), spleen (**B**), thymus (**C**), ovary (**F**), and uterus (**G**), indicating that restraint stress affects the immune system, the digestive system, and the reproductive system. At the same time, the heart (**D**) index and kidney (**E**) index increased under restraint stress. In general, restraint stress may affect the function of the various systems of the body. The data were expressed as the mean ± SEM. Non-Pregnancy Day 5 (NP5) (n_stress_ = 10, n_control_ = 10), Pregnancy Day 5 (P5) (n_stress_ = 10, n_control_ = 10), Pregnancy Day 10 (P10) (n_stress_ = 10, n_control_ = 10), Pregnancy Day 15 (P15) (n_stress_ = 10, n_control_ = 10), and Pregnancy Day 20 (P20) (n_stress_ = 10, n_control_ = 10). Lowercase letters in the column (a, b, c) represented the differences between NP5, P5, P10, P15, and P20 in the control group (*p* < 0.05), and uppercase letters in the column (A, B, C, D) represented the differences between NP5, P5, P10, P15, and P20 in the stress group (*p* < 0.05). * *p* < 0.05 and # *p* < 0.01 were used to denote the significance of the stress group compared with the corresponding control group.

In this study, there was no apparent pathological change in most organs, according to the pathologic anatomy (Figure S1). Thus, we measured the weights of the liver, spleen, thymus, heart, kidney, ovary, and uterus. The organ weight and body weight ratio represent the organ index. From Figure 2, the pregnancy age affected the change in all organ indexes (*p* <0.001) and the organ indexes were affected by restraint stress (Liver, *p* < 0.001; spleen, *p* < 0001; thymus, *p* < 0.001; heart, *p* < 0.001, kidney; *p* < 0.001; ovary, *p* = 0.005; uterus, *p* = 0.009). The indexes of the liver (independent sample *t* test, NP5: 9.62%, *p* = 0.013; P5: 16.10%, *p* = 0.008; P15: 8.41%, *p* = 0.010; P20: 7.35%, *p* = 0.048) (Figure 2A), spleen (independent sample *t* test, P5: 26.74%, *p* = 0.008; P10: 21.79%, *p* = 0.034; P15: 32.81%, *p* = 0.001; P20: 14.06%, *p* = 0.045) (Figure 2B), thymus (independent sample *t* test, NP5: 40.86%, *p* < 0.001; P5: 46.17%, *p* < 0.001; P10: 53.64%, *p* < 0.001; P15: 41.79%, *p* = 0.005; P20: 19.62%, *p* = 0.023) (Figure 2C), ovary (independent sample *t* test, NP5: 25.87%, *p* = 0.008; P10: 16.50%, *p* = 0.016), and uterus (independent sample *t* test, NP5: 24.10%, *p* < 0.001; P5: 24.51%, *p* = 0.016; P20: 20.67%, *p* = 0.047) decreased significantly in the stress group compared with the control group (Figure 2F,G). The indexes had the same tendency in the control and stress groups with changes in age (Figure 2A–C,F,G). However, the indexes of the heart (independent sample *t* test, P15: 44.75%, *p* = 0.003; P20: 23.76%, *p* = 0.008) and kidney (Independent sample *t* test, P15: 24.46%, *p* = 0.019; P20: 16.76%, *p* = 0.003) were obviously increased in stressed mice compared with control mice at different ages (Figure 2D,E) (for the specific value, see Table S2 of Supplementary Material). In summary, restraint stress may affect the index of organ.

### 2.3. Effect of Restraint Stress on Plasma Hormone Levels in Pregnant Mice

**Figure 3:**
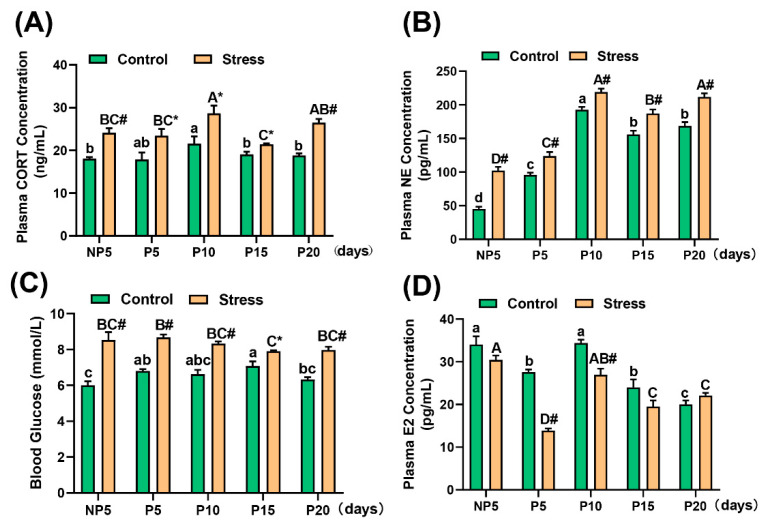
Changes in plasma hormone levels in pregnant mice. Restraint stress caused an increase in corticosterone (CORT) (**A**), norepinephrine (NE) (**B**), and blood glucose (**C**) levels but a decrease in oestradiol (E2) (**D**) in the plasma of pregnant mice. Restraint stress caused disorders of stress hormones and reproductive hormone levels, resulting in endocrine disorders. The data are expressed as the mean ± SEM. Lowercase letters in the column (a, b, c, d) represent the differences between NP5 (*n* = 10), P5 (*n* = 10), P10 (*n* = 10), P15 (*n* = 10), and P20 (*n* = 10) in the control group (*p* < 0.05), and uppercase letters in the column (A, B, C and D) represent the differences between NP5 (*n* = 10), P5 (*n* = 10), P10 (*n* = 10), P15 (*n* = 10), and P20 (*n* = 10) in the stress group (*p* < 0.05). * *p* < 0.05 and # *p* < 0.01 were used to denote the significance of the stress group compared with the corresponding age of the control group.

As is known, stress activates the hypothalamic–pituitary–adrenal (HPA) axis and the sympathetic-adrenal medullary system (SAS), which induce the release of glucocorticoids from the adrenal cortex and the catecholamines epinephrine (EPI) and NE from the adrenal medulla and sympathetic nerve termini [22]. In our study, the concentration of stress hormones increased significantly, including the concentrations of CORT (independent sample *t* test, NP5: 6.048 ng/mL, 33.48%, *p* = 0.001, P5: 3.544 ng/mL, 17.82%, *p* = 0.044, P10: 7.118 ng/mL, 33.05%, *p* = 0.040; P15: 2.411 ng/mL 12.67%, *p* = 0.010; P20: 7.669 ng/mL, 40.82%, *p* < 0.001) (Figure 3A) and NE (independent sample *t* test, NP5: 56.754 pg/mL, 125.01%, *p* < 0.001; P5: 28.205 pg/mL, 29.49%, *p* < 0.001; P10: 26.251 pg/mL, 13.61%, *p* < 0.001; P15: 31.445 pg/mL, 20.18%, *p* < 0.001; P20: 43.289 pg/mL, 25.68%, *p* < 0.001) (Figure 3B). Additionally, gluconeogenesis is usually enhanced by glucocorticoids and then increases blood glucose levels [23]. Compared with control mice, in stress mice, the blood glucose level increased significantly in each age group (independent sample *t* test, NP5: 2.533 mmol/L, 42.22%, *p* = 0.007; P5: 1.875 mmol/L, 27.57%, *p* < 0.001; P10: 1.700 mmol/L, 25.63%, *p* = 0.003; P15: 0.825 mmol/L, 11.66%, *p* = 0.047; P20: 1.633 mmol/L, 25.79%, *p* = 0.002) (Figure 3C). As is well known, E2 is a marker of female reproduction. The concentration of E2 was detected by radioimmunoassay, and there was a significant decrease at P5 (independent sample *t* test, 13.704 pg/mL, 49.66%, *p* < 0.001) and P10 (independent sample *t* test, 7.440 pg/mL, 21.63%, *p* = 0.004) in stress mice compared with control mice (Figure 3D) (for the specific value, see Table S3 of Supplementary Material). A two-way analysis of variance showed that the age of pregnancy affected the change in these index levels (*p* < 0.001), except for the blood glucose (*p* = 0.085), and all of them were influenced by restraint stress (*p* < 0.001). This result showed that restraint stress increased stress levels and inhibited E2 levels.

### 2.4. Effect of Restraint Stress on Antioxidative Ability in the Uterus of Pregnant Mice

**Figure 4:**
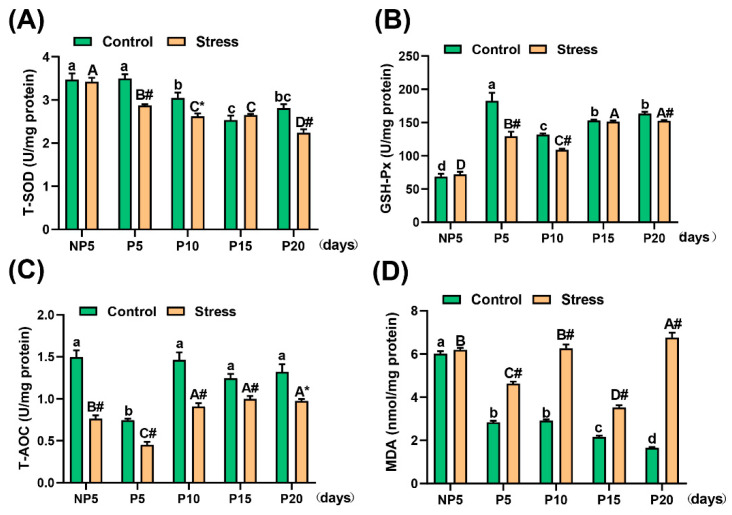
Effects of restraint stress on the oxidative stress states in the uterus in pregnant mice. These graphs show the contents of total superoxide dismutase (T-SOD) (**A**), glutathione peroxidase (GSH-Px) (**B**), total antioxidant capacity (T-AOC) (**C**), and malondialdehyde (MDA) (**D**) in the uterus of the control and stress group at NP5, P5, P10, P15, and P20. Restraint stress reduced the levels of T-SOD (**A**), GSH-Px (**B**), and total antioxidant capacity (**C**) in pregnant mice, resulting in increased lipid peroxide MDA content (**D**). In conclusion, restraint stress reduced the antioxidant capacity of the uterus in pregnant mice. Values are expressed as the mean ± SEM. Lowercase letters in the column (a, b, c, d) represent the differences between NP5 (*n* = 10), P5 (*n* = 10), P10 (*n* = 10), P15 (*n* = 10), and P20 (*n* = 10) in the control group (*p* < 0.05), and uppercase letters in the column (A, B, C and D) represent the differences between NP5 (*n* = 10), P5 (*n* = 10), P10 (*n* = 10), P15 (*n* = 10), and P20 (*n* = 10) in the stress group (*p* < 0.05). * *p* < 0.05 and # *p* < 0.01 were used to denote the significance of the stress group compared with the corresponding control group.

Antioxidant enzymes and oxidative products represent the antioxidative ability and oxidant level of mice, respectively. The related anti-oxidases were tested, including Glutathione peroxidase (GSH-Px), T-SOD, and Total Antioxidant Capacity (T-AOC). The concentrations of T-SOD and GSH-Px in the stress group compared with the control group were significantly decreased at P5 (independent sample *t* test, T-SOD, 0.626 U/mg, 17.89%, *p* < 0.001; GSH-Px, 53.409 U/mg, 29.23%, *p* = 0.001), P10 (independent sample *t* test, T-SOD, 0.427 U/mg, 14.02%, *p* = 0.011; GSH-Px, 23.180 U/mg, 17.57%, *p* < 0.001), and P20 (independent sample *t* test, T-SOD, 0.576 U/mg, 20.49%, *p* < 0.001; GSH-Px, 10.956 U/mg, 6.70%, *p* < 0.001) (Figure 4A,B). The activity of T-AOC decreased significantly in the stress group compared with the control group at every age (independent sample *t* test, NP5: 0.735 U/mg, 49.10%, *p* < 0.001; P5: 0.294 U/mg, 39.52%, *p* < 0.001; P10: 0.556 U/mg, 37.98%, *p* < 0.001; P15: 0.247 U/mg, 19.84%, *p* = 0.001; P20: 0.347 U/mg, 26.27%, *p* = 0.012) (Figure 4C). Furthermore, the levels of Malondialdehyde (MDA) increased in each age group except NP5. Additionally, the degree of elevation was significant (independent sample *t* test, P5: 1.798 nmol/mg, 63.43%, *p* < 0.001; P10: 3.348 nmol/mg, 114.97%, *p* < 0.001; P15: 1.363 nmol/mg, 63.12%, *p* < 0.001; P20: 5.101 nmol/mg, 307.84%, *p* < 0.001) (Figure 4D) (for the specific value, see Table S4 of Supplementary Material). A two-way analysis of variance found that both pregnancy age (*p* < 0.001) and restraint stress (*p* < 0.001) affected the change in anti-oxidation ability. Overall, the antioxidative ability of the uterus decreased under restraint stress.

### 2.5. Effect of Restraint Stress on FOXO1, FOXO3, and FOXO4 mRNA Levels and FOXO1 Protein in the Uterus and Ovary of Pregnant Mice

**Figure 5:**
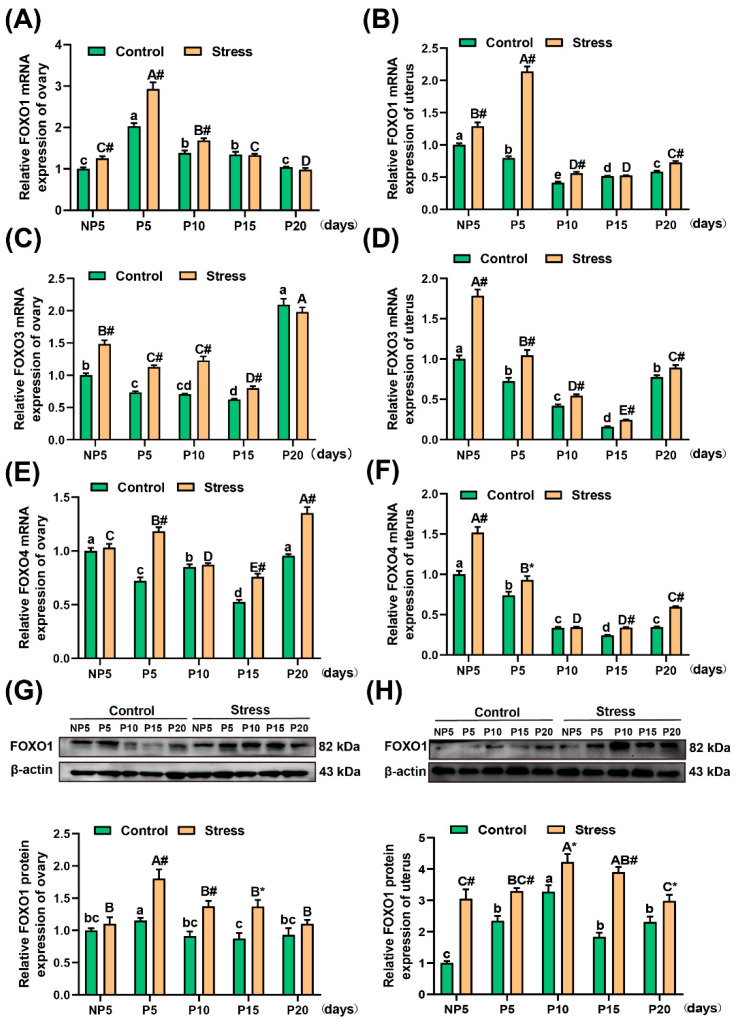
Effect of restraint stress on FOXO mRNA levels and FOXO1 protein in the uterus and ovary of pregnant mice. Restraint stress significantly increased the level of Forkhead transcription factor class O (FOXO) mRNA associated with oxidative stress in the ovary (**A**,**C**,**E**) and uterus (**B**,**D**,**F**), suggesting that the oxidative stress experienced by the body was excessive. Furthermore, the relative expression of FOXO1 was highest among FOXO, so the protein level of FOXO1 was detected by WB and significantly increased in the ovary (**G**) and uterus (**H**). The data are expressed as the mean ± SEM. Lowercase letters in the column (a, b, c and d) represent the differences between NP5 (*n* = 10), P5 (*n* = 10), P10 (*n* = 10), P15 (*n* = 10), and P20 (*n* = 10) in the control group (*p* < 0.05), and uppercase letters in the column (A, B, C, D and E) represent the differences between NP5 (*n* = 10), P5 (*n* = 10), P10 (*n* = 10), P15 (*n* = 10), and P20 (*n* = 10) in the stress group (*p* < 0.05). The value of NP1 was regard as 1 to be used as the base level of assessment. * *p* < 0.05 and # *p* < 0.01 were used to denote the significance of the stress group compared with the corresponding control group.

The FOXO family plays an important role in oxidative stress and reproduction. In this study, FOXO family members were studied via real-time fluorescence quantitative PCR (qRT-PCR) and Western blotting. As shown in Figure 5, in the stress group, the mRNA levels of FOXO1 in the ovary increased significantly at NP5 (independent sample *t* test, 25.38%, *p* < 0.001), P5 (independent sample *t* test, 44.29%, *p* < 0.001), and P10 (independent sample *t* test, 22.11%, *p* < 0.001) (Figure 5A). In the uterus, the mRNA levels of FOXO1 increased significantly at NP5 (independent sample *t* test, 29.01%, *p* < 0.001), P5 (independent sample *t* test, 168.07%, *p* < 0.001), P10 (independent sample *t* test, 36.04%, *p* < 0.001), and P20 (independent sample *t* test, 24.45%, *p* < 0.001) (Figure 5B). Furthermore, the mRNA levels of FOXO3 increased significantly at NP5 (independent sample *t* test, 48.53%, *p* < 0.001), P5 (independent sample *t* test, 53.89%, *p* < 0.001), P10 (independent sample *t* test, 73.64%, *p* < 0.001), and P15 (independent sample *t* test, 27.99%, *p* < 0.001) in the ovary (Figure 5C). These levels also increased significantly at NP5 (independent sample *t* test, 78.34%, *p* < 0.001), P5 (independent sample *t* test, 43.81%, *p* = 0.001), P10 (independent sample *t* test, 30.08%, *p* < 0.001), P15 (independent sample *t* test, 53.52%, *p* < 0.001), and P20 (independent sample *t* test, 15.07%, *p* = 0.006) in the uterus (Figure 5D) (for the specific value, see Table S5 of Supplementary Material).

Additionally, the changes seemed to be the same for FOXO4 by FOXO1 and FOXO3. The mRNA levels of FOXO4 in the ovary increased in each age group, especially at P5 (independent sample *t* test, 63.67%, *p* < 0.001), P15 (independent sample *t* test, 44.14%, *p* < 0.001), and P20 (independent sample *t* test, 41.77%, *p* < 0.001) (Figure 5E). Similarly, the mRNA levels of FOXO4 in the uterus increased significantly at NP5 (independent sample *t* test, 51.74%, *p* < 0.001), P5 (independent sample *t* test, 25.98%, *p* = 0.016), P15 (independent sample *t* test, 38.42%, *p* < 0.001), and P20 (independent sample *t* test, 73.09%, *p* < 0.001) (Figure 5F) (for the specific value, see Table S5 of Supplementary Material). A two-way analysis of variance showed that both pregnancy age (*p* < 0.001) and restraint stress (*p* < 0.001) affected the change in mRNA levels of FOXO1, FOXO3 and FOXO4.

From the Table S5, the mRNA expression of FOXO1 were higher than FOXO3 and FOXO4. Meanwhile, FOXO1 is more involved in the stress and reproduction than FOXO3 and FOXO4. Thus, FOXO1 protein expression was detected in the ovary and uterus. A two-way analysis of variance showed that both pregnancy age (*p* < 0.001) and restraint stress (*p* < 0.001) affected the change in protein levels of FOXO1. The protein expression of FOXO1 was obviously increased at P5 (independent sample *t* test, 56.57%, *p* = 0.005), P10 (independent sample *t* test, 50.66%, *p* = 0.001) and P15 (independent sample *t* test, 57.18%, *p* = 0.010) in the ovary (Figure 5G). Furthermore, in the uterus, the protein expression of FOXO1increased significantly at NP5 (independent sample *t* test, 204.72%, *p* = 0.003), P5 (independent sample *t* test, 40.50%, *p* < 0.001), P10 (independent sample *t* test, 28.75%, *p* = 0.011), P15 (independent sample *t* test, 112.56%, *p* < 0.001), and P20 (independent sample *t* test, 28.87%, *p* = 0.035) (Figure 5H) (for the specific value, see Table S5 of Supplementary Material). Overall, the levels of FOXO1, FOXO3, and FOXO4 increased in the stress group.

### 2.6. Effect of Restraint Stress on β_2_-AR Levels in the Uterus and Ovary of Pregnant Mice

**Figure 6:**
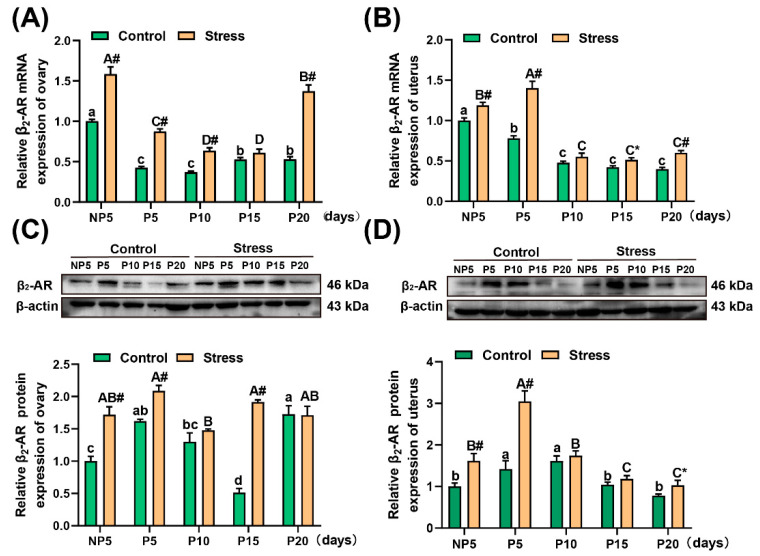
Effect of restraint stress on β_2_-AR in the uterus and ovary of pregnant mice. Oxidative stress led to a significant increase in mRNA levels and protein expression of β_2_-AR in the ovary (**A**,**C**) and uterus (**B**,**D**) of pregnant mice, suggesting that oxidative stress caused the body’s G-protein coupled receptors to be activated. The mRNA levels of β_2_-AR were detected by quantitative real-time polymerase chain reaction (qRT-PCR), and the protein level of β_2_-AR was measured by WB. The data are expressed as the mean ± SEM. Lowercase letters in the column (a, b, c, d) represent the differences between NP5 (*n* = 10), P5 (*n* = 10), P10 (*n* = 10), P15 (*n* = 10), and P20 (*n* = 10) in the control group (*p* < 0.05), and uppercase letters in the column (A, B, C and D) represent the differences between NP5 (*n* = 10), P5 (*n* = 10), P10 (*n* = 10), P15 (*n* = 10), and P20 (*n* = 10) in the stress group (*p* < 0.05). The value of NP1 was regard as 1 to be used as the base level of assessment. * *p* < 0.05 and # *p* < 0.01 were used to denote the significance of the stress group compared with the corresponding control group.

In this study, a two-way analysis of variance showed that the age of pregnancy (*p* < 0.001) and restraint stress (*p* < 0.001) affected the change in mRNA and protein levels of β_2_-AR. The mRNA levels of β_2_-AR increased significantly at NP5 (independent sample *t* test, 58.43%, *p* < 0.001), P5 (independent sample *t* test, 105.72%, *p* < 0.001), P10 (independent sample *t* test, 71.90%, *p* < 0.001), and P20 (independent sample *t* test, 159.03%, *p* < 0.001) in the ovary (Figure 6A); in the uterus, the mRNA levels of β_2_-AR increased significantly at NP5 (independent sample *t* test, 18.84%, *p* = 0.002), P5 (independent sample *t* test, 79.62%, *p* < 0.001), P15 (independent sample *t* test, 21.68%, *p* = 0.011), and P20 (independent sample *t* test, 50.92%, *p* < 0.001) (Figure 6B). Additionally, the protein expression of β_2_-AR was detected in this study. As shown in Figure 6, the protein expression of β_2_-AR in the ovary was obviously increased in each age group (independent sample *t* test, NP5: 71.97%, *p* = 0.002; P5: 28.93%, *p* = 0.006; P15: 272.57%, *p* < 0.001) (Figure 6C). In the uterus, the protein levels of β_2_-AR increased significantly at NP5 (independent sample *t* test, 61.87%, *p* = 0.007), P5 (independent sample *t* test, 114.72%, *p* = 0.005), and P20 (independent sample *t* test, 32.31%, *p* = 0.031) (Figure 6D) (for the specific value, see Table S6 of Supplementary Material). The stimulation of restraint stress caused a significant increase in the protein expression of β_2_-AR. Overall, the expression of β_2_-AR increased under restraint stress.

### 2.7. Immunohistochemical Localization of FOXO1

**Figure 7:**
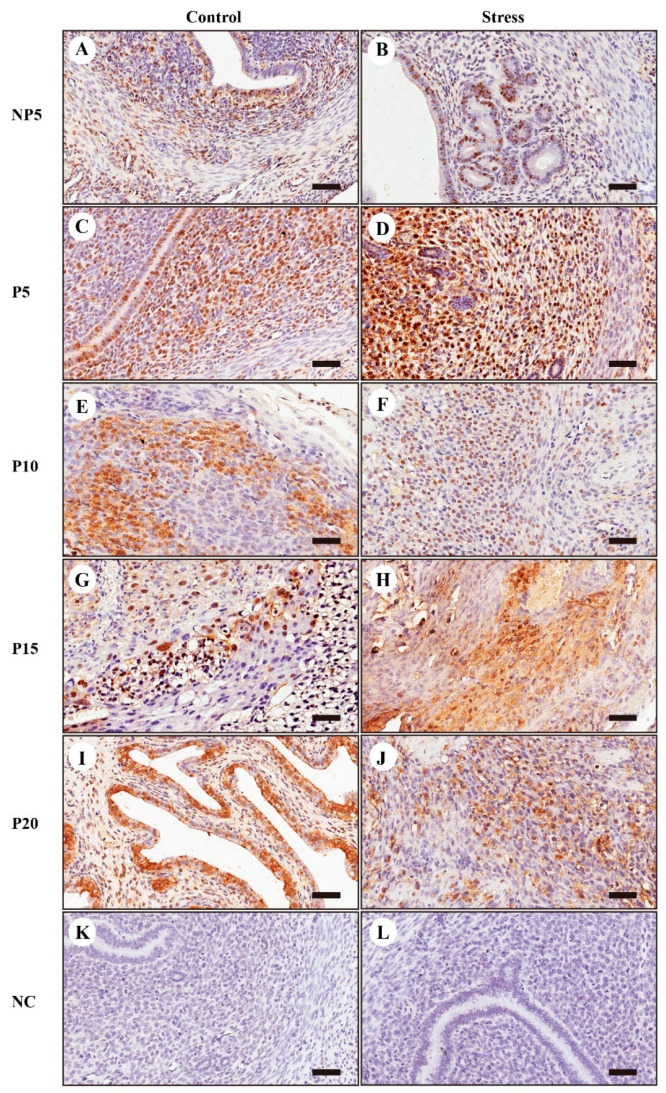
Immunohistochemical localization of FOXO1. To detect FOXO1 expression in the uterus, immunohistochemical localization was performed. A large number of positive cells were expressed in endometrial stromal cells of the uterus at NP5 (**A**,**B**), P5 (**C**,**D**), P10 (**E**,**F**), P15 (**G**,**H**), and P20 (**I**,**J**). The negative control (NC) were **K** (Control group) and **L** (Stress group). The yellow particles indicate positive cells. The scale bar is 50 μm.

To explore the relationship between β_2_-AR and FOXO1, we performed immunohistochemical localization of FOXO1 in the uterus at every gestation age. The brownish yellow portion indicated positive cells. At NP5, P5, P10, P15 and P20, the FOXO1 was located in the endometrial luminal epithelium cell and in the ESCs. Meanwhile, FOXO1 was located at the nuclear during pregnancy, which demonstrated that FOXO1 exerted the transcription regulation function during pregnancy. As shown in Figure 7 and Figure 5H, at P5, FOXO1 increased under restraint stress, which showed that FOXO1 was not only involved in pregnancy but also related to restraint stress.

### 2.8. Relativity Analysis among FOXO1, p-NF-κB p65, and β_2_-AR

**Figure 8:**
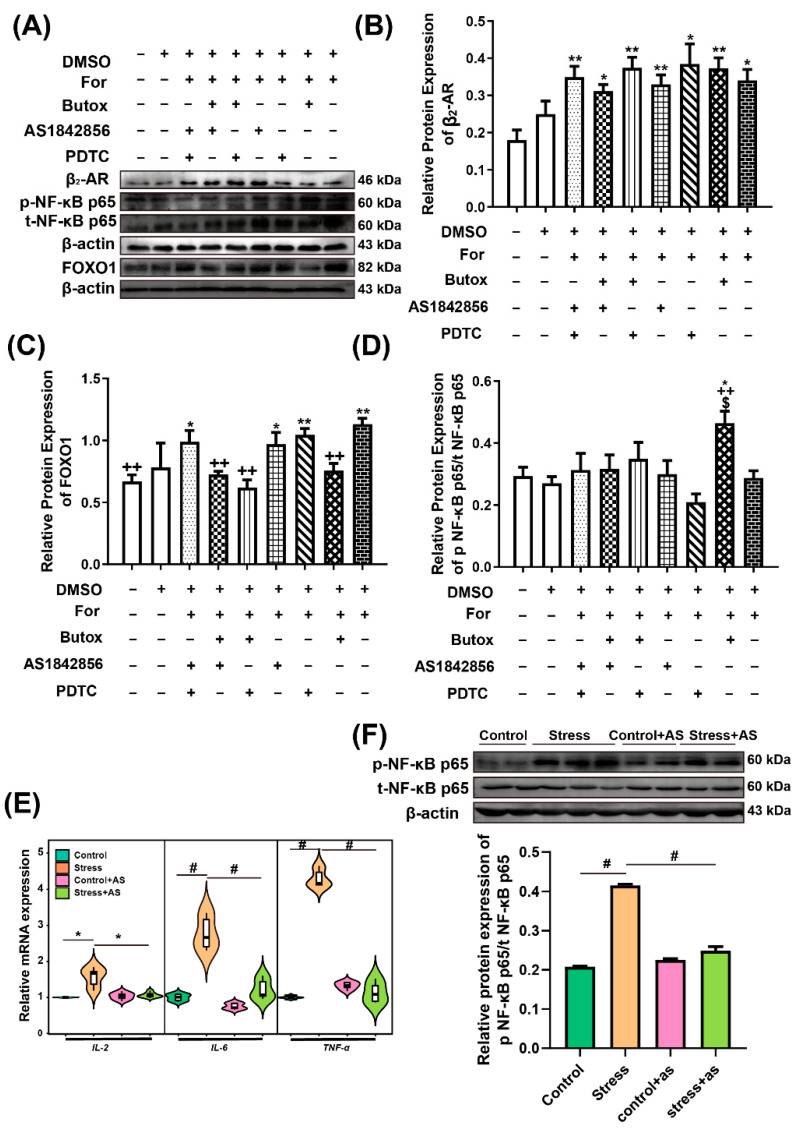
β_2_-AR activates FOXO1 and FOXO1 induced the expression of p-NF-κB p65/t-NF-κB p65 protein and its downstream inflammation genes. These protein bands of β_2_-AR, FOXO1, p-NF-κB p65, and t-NF-κB p65 in ESCs were tested by adding agonist and blocking agents (**A**). The protein levels of β_2_-AR increased after adding the agonists of β_2_-AR compared with the control cells (**B**). Interestingly, the protein levels of FOXO1 were obviously increased when β_2_-AR was activated but were significantly decreased when Butox was added to the cultured cells (**C**). It is worth noting that the protein expression of p-NF-κB p65/t-NF-κB p65 did not change when β_2_-AR was activated, but it increased when β_2_-AR was blocked by Butox, and it decreased significantly when FOXO1 was inhibited by AS1842865 (**D**). (**E**) The mRNA levels of IL-2, IL-6, and TNF-α in the uterus of pregnant mice at P5 after restraint stress and adding AS. (**F**) Protein band and quantitative analysis of p-NF-κB p65/t-NF-κB p65. Formoterol hemifumarate (For) is a selective agonist of β_2_-AR. Butox is the blocking agent of β_2_-AR, and AS1842865 is an inhibitor of FOXO1. Pyrrolidine dithiocarbamate ammonium (PDTC) is an inhibitor of NF-κB. DMSO is the solvent for the drugs. These data are expressed as the mean ± SEM. * *p* < 0.05 and ** *p* < 0.01 were used to denote the significance of the other groups compared with the corresponding control group (**B**). ++ *p* < 0.01 were used to denote the significance of other groups compared with the corresponding For group. $ *p* < 0.05 was used to denote the significance of other groups compared with the cell+For+Butox+AS group. Independent sample *t* test was performed, * *p* < 0.05 and # *p* < 0.01 were used to denote the significance of the stress group compared with the corresponding control group (**E** and **F**).

In view of the above, we isolated and cultured the ESCs of the uterus at P5 to explore the relationship between FOXO1, p-NF-κB p65, and β_2_-AR. The protein levels of β_2_-AR increased after adding the agonists of β_2_-AR compared with control cells (independent sample *t* test, 88.81%*, p* = 0.010) (Figure 8A,B). Interestingly, the protein levels of FOXO1 were also obviously increased when β_2_-AR was activated (independent sample *t* test 69.19%, *p*= 0.001) (Figure 8A,C). However, the protein levels of FOXO1 decreased significantly when Butoxamine hydrochloride (Butox) was added to the cultured cells (independent sample *t* test, 33.14%, *p* = 0.001) (Figure 8A,C). It is worth noting that the protein expression of p-NF-κB p65 did not change when β_2_-AR was activated, but it increased obviously when β_2_-AR was blocked by Butox (independent sample *t* test, 61.64%, *p* = 0.009) (Figure 8A,D). Furthermore, the expression decreased significantly when FOXO1 was inhibited by AS1842865 (independent sample *t* test, 47.53%, *p* = 0.022) compared with Butox treatment (Figure 8A,D). Next, in vivo, the target gene of p-NF-κB p65, including IL-2 (independent sample *t* test, 44.94%, *p* = 0.021), Interleukin-6 (IL-6) (independent sample *t* test, 177.14%, *p* = 0.001), and the target gene of FOXO1, tumour necrosis factor-alpha (TNF-α) (independent sample *t* test, 322.70%, *p* < 0.001), increased significantly in the restraint stress group compared with control group. However, they decreased when the FOXO1 was inhibited in the restraint stress group (independent sample *t* test, IL-2, 30.25%, *p* = 0.032; IL-6, 55.95%, p = 0.001; TNF-α, 73.08%, *p* < 0.001) (Figure 8E) compared with restraint stress group. Meanwhile, the protein levels of p-NF-κB p65/t- NF-κB p65 were obviously facilitated (independent sample *t* test, 99.77%, *p* < 0.001) in the restraint stress group. However, it decreased when the FOXO1 was inhibited (independent sample *t* test, 40.09%, *p* < 0.001) (Figure 8F) (for the specific value, see Table S7 of Supplementary Material). Therefore, FOXO1 may be a critical mediator in the crosstalk between β_2_-AR and p-NF-κB p65 in ESCs.

## 3. Discussion

### 3.1. Restraint Stress Disturbs Neuroendocrine Signalling in Pregnant Mice

Stress can cause neuroendocrine disorders and a range of reproductive diseases. For example, long-term chronic prenatal stress can lead to postpartum depression [24], pregnancy obesity syndrome [25], endometriosis, cancer, recurrent pregnancy loss, and pregnancy complications associated with pre-term birth [26]. The mechanism of stress on reproduction involved the HPA and hypothalamic–pituitary–gonadal (HPG) axis. Restraint stress can induce psychological stress, which induces the activation of the HPA axis [27]. Activation of the HPA axis by various stressors primarily inhibits reproductive function and is able to alter foetal development, inducing biological stress in the uterus [28]. CORT, NE, and blood glucose levels in mice are generally regarded as stress makers [29,30]. CORT can reduce the amount of oestradiol in the body and can cause damage during the reproductive process [31]. Therefore, restraint stress exacerbates this negative effect during pregnancy by activating the HPA axis. In this study, CORT, NE, and blood glucose levels increased significantly in stress mice, and E2 concentration decreased significantly at P5 and P15 (Figure 3). Disordered E2 affects eating, energy expenditure, and body adiposity [32]. Meanwhile, anxiety can induce weight loss [33]. In this study, the loss of body weight significantly increased after restraint stress at different ages compared with the control group. Elevation of CORT and NE caused by restraint stress reduced the spleen index in mice and induced an involution of red pulp and an expansion of white pulp. This phenomenon is also involved in the alterations of spleen immune cell subsets [34]. In summary, we believe that restraint stress disrupted hormone levels and resulted in malnutrition during pregnancy.

It was well known that E2 is important to embryo implantation and supports the growing foetus and counteract pregnancy stresses [35]. Generally, oxidative stress is accompanied by a decrease in E2 levels, and E2 treatment can maintain the redox balance. Therefore, oxidative stress is one of the reasons for the decrease in E2 levels [36]. Related studies have shown that oxidative stress can cause a variety of reproductive diseases, including endometriosis [37,38], foetal growth restriction [39], and intrauterine immune disorders [13,40]. In this work, restraint stress induced the oxidative stress. T-AOC level decreased at NP5 and throughout pregnancy (P5-P20) under restraint stress. In particular, T-SOD and GSH-Px decreased in the implantation (P5), post-implantation (P10), and childbirth periods (P20). The levels of MDA were significantly increased throughout pregnancy (P5-P20) (Figure 4). SOD, T-AOC, and GSH-Px, biomarkers of antioxidation, are decreased under oxidative stress. MDA, a biomarker of oxidative stress reflected by oxidation level, is increased under oxidative stress [41]. Stress not only stimulates the HPA axis but also disrupts the balance between oxidative and antioxidative stress [42]. Therefore, we considered that restraint stress threatened the intracellular equilibrium between oxidants and antioxidants.

Oxidative stress is closely related to the immune response. The indexes of the spleen and thymus, the lymphatic organs used in this study, decreased in stressed mice, showing that restraint stress leads to immune disorders in pregnant mice (Figure S1 and Figure 2). Our previous study suggested that restraint stress disturbed the balance of the uterine immune microenvironment during the implantation period, which altered the number of uterus natural killer cell (uNK) and mast cells, decreased the CD3+CD4+ T/CD3+CD8+ T cell ratio and the proliferative activities of T and B lymphocytes, and increased the IL-2/IL-4 ratio [5]. In our study, the uterus index was also decreased in the implantation (P5) and childbirth periods (P20) (Figure S1 and Figure 2). Therefore, we speculated that restraint stress may disrupt the immune environment of pregnant mothers and may limit uterine cell proliferation.

### 3.2. The FOXO Family is Involved in the Oxidative Stress Induced by Restraint Stress

FOXO, an evolutionarily conserved subfamily of Forkhead transcription factors, has emerged as a master regulator of cell fate decisions capable of integrating a variety of stress, growth factor and cytokine signalling pathways with the transcription machinery [43]. FOXO transcription factors function mostly as transcriptional activators, and their activity is inhibited by the phosphatidylinositol 3-kinase-serine/threonine kinase (PI3K-AKT) signalling pathway, resulting in FOXO exclusion from the nucleus and repression of transcriptional activity in order to resist oxidative stress [44]. There have also been reports that FOXO ablation in myeloid cells increases the generation of ROS [45], and that FOXO1 enhances the antioxidant capacity under normal physiological conditions [30]. Treatment with either FOXO1 small interfering RNA (siRNA) or resveratrol, a sirt1 agonist, results in oxidative stress in QBC939 cells [46]. FOXO also plays a critical role in the induction of human uterine decidualization [47]. The capabilities of trophoblast adhesion and migration are inhibited by silencing FOXO1 or oxidative stress with H_2_O_2_ [48].

In this work, mRNA levels of FOXO1, FOXO3, and FOXO4 were detected in the uterus and ovary, and all of them were significantly elevated under restraint stress during pregnancy. As stated previously, it is well known that FOXO1, FOXO3, and FOXO4 differ only in tissue specificity. FOXO1, FOXO3, and FOXO4 proteins are localized in the cytoplasm during embryo development and are located in the nucleus in embryos with developmental delay [49]. In the setting of growth factor stimulation, FOXO1, -3, and -4 are subject to Akt-mediated phosphorylation, which results in their nuclear export and the inhibition of FOXO-mediated transcription [44]. Therefore, we performed a protein assay of FOXO1 to illustrate the effect of restraint stress on the FOXO family. Interestingly, although FOXO family members, especially FOXO1, were activated by restraint stress, the antioxidation levels decreased in the stress group. Similar reports, in which a vicious cycle of oxidative stress results in insulin resistance (InsRes), promoting sustained FOXO activity and the induction of apoptosis, have been made [50]. Oxidative stress may promote the transcriptional activity of FOXO proteins, resulting in hyperglycaemia and a further increased production of ROS [51]. Therefore, the decline in antioxidant capacity confirms the excessive increase in ROS, indicating that ROS are promoted by FOXO and result in a vicious cycle.

Overall, there are two sides to the FOXO response to oxidative stress. Short-term stress promotes the upregulation of protective mechanisms. However, oxidative stress is not addressed, and associated damage is continuously activated. As a result, FOXO may lead to an induction of apoptosis. For instance, FOXO1 is a positive regulator of bone formation by resisting oxidative stress and favouring protein synthesis in osteoblasts [52]. However, continuously active FOXO1 overexpression induces apoptosis associated with the altered expression of genes regulating cell cycle and survival in rheumatoid arthritis fibroblast-like synoviocytes [53].

### 3.3. Restraint Stress Promotes the β_2_-AR Pathway

β_2_-AR belongs to the G protein-coupled receptor. It promotes or inhibits the expression of cyclic adenosine monophosphate (cAMP) in combination with Gs [54] and Gi [55,56]. β_2_-AR is closely related to the secretion of oestradiol and progesterone in mothers during pregnancy, and it may be involved in the process of embryo implantation [57]. As is well known, endogenous catecholamines such as EPI and NE are released from the adrenal gland and sympathetic nervous system under stress. The adrenergic system plays a central role in stress signalling, and excessive stress has been found to be associated with increased production of ROS [58].

NE and EPI stimulate cell surface α- (α_1A_, α_1B_, α_1D_, α_2A_, α_2B_, and α_2C_) and β- (β_1_, β_2_, and β_3_) adrenergic receptors (ARs) with differing affinities [59]. The expression of adrenergic receptor mRNA in the uterus of E4 mice has been detected using reverse transcription-polymerase chain reaction (RT-PCR), and it was found that the expression of β_2_-AR mRNA was much higher than in other subtypes [9]. In our study, the mRNA levels and protein expression levels of β_2_-AR increased significantly in the ovary and uterus under restraint stress. This implied that restraint stress activated β_2_-AR (Figure 6). β_2_-AR often mediates intracellular and extracellular signalling during stress [60]. Sympathetic nervous system activation and immune suppression occur due to the chronic stress of caregiving. β_2_-AR contributes to the stress-induced loss of immune-cell function [61]. Stress-induced elevation of EPI levels results in the activation of β_2_-AR on keratinocytes [62]. A study showed that β_2_-AR mediated the effect of chronic stress on lesions and exacerbated endometriosis-associated generalized hyperalgesia, thus accelerating the development of endometriosis [63]. In the development of pancreatic cancer, psychological stress is considered to be a risk factor associated with β_2_-AR [64].

### 3.4. FOXO1 Mediates the β_2_-AR and p-NF-κB p65 Signalling Pathways

In vivo studies have shown that FOXO1 and β_2_-AR undergo significant changes under restraint stress conditions. In current studies, we investigated whether FOXO1 and β_2_-AR mediated NF-κB p65. The immunohistochemical results showed that FOXO1 was expressed in ESCs at every gestation age, so this study selected ESCs to explore signal transduction (Figure 7).

In recent years, the relationship between β_2_-AR and NF-κB has been studied by a large number of researchers, and many achievements have been made in inflammation and apoptosis. In this work, we focused on the relationship between β_2_-AR, FOXO1, and NF-κB; we also added a selective agonist of β_2_-AR (formoterol hemifumarate), which activates the intracellular signalling pathway, and added a blocker or inhibitor of β_2_-AR (butox), FOXO1 (AS1842865), and NF-κB (PDTC) to explore the relationship between the three factors. However, we found a more interesting phenomenon in cultured cells in vitro. Although FOXO1 was activated with formoterol hemifumarate, the phosphorylation of NF-κB p65 did not change. This was consistent with related reports. In some studies, β_2_-AR inhibited the degradation of inhibitor of NF-κB (IκB) [65,66,67], thereby impeding NF-κB entry into the nucleus and reducing inflammation [68,69]. This was related to β-arrestin 2, which prevents the inflammation of NF-κB p65 [70]. Gao et al. proved that β_2_-AR promoted IκB binding to NF-κB p65 to prevent it from entering the nucleus in order to increase the expression of IL-6, interleukin-8 (IL-8), tumour necrosis factor-alpha (TNF-α), and interleukin-1 beta (IL-1β) [71]. However, many studies have suggested that isoproterenol (ISO) activates NF-κB DNA binding activity and induces myocardial and systemic elaboration of interleukin-18 (IL-18) via β_2_-AR signalling, thereby promoting inflammation [72,73]. These results were different from our conclusion, which was considered to be due to the different agonists used. These previous studies used ISO (non-selective of β_2_-AR), but we used formoterol hemifumarate (a specific agonist of β_2_-AR). The difference might also be caused by pathways other than β_2_-AR.

Notably, when butox, the blocking agent of β_2_-AR, was added, the phosphorylation of NF-κB p65 was significantly increased. We speculated that there may be another way to activate p-NF-κB p65. Freddolino et al. reported that β_2_-AR had a number of residues for agonist and antagonist binding, including Asp-113; Ser-203, -204, and -207, Asn-293, Ile-169, Val-117, and Phe-290. Butox acted as an antagonist interacting with Ser-203, Ser-204, Asn-293, and Phe-290 of β_2_-AR and was considered to be a strong β_2_-AR-selective antagonist because of its strong binding to Asn-293, but it did not bind to the more potent Ser-207 [74]. Therefore, we speculated that the Butox may only block the site that can activate β-arrestin 2 to promote the activity of NF-κB. Thus, it made the formoterol hemifumarate able to activate p-NF-κB p65. The mechanism is unclear in current studies. Therefore, we speculated that β_2_-AR may also have promoted the increase in NF-κB to a certain extent.

In this work, it was found that β_2_-AR could significantly activate the protein levels of FOXO1. Furthermore, a study reported that administration of β_2_-AR agonists in mouse skeletal muscle induced expression of FOXO1 [21]. Both insulin resistance and oxidative stress may promote the transcriptional activity of FOXO proteins, resulting in a further increased production of ROS [51]. However, Zhang et al. proved that β_2_-AR was able to mediate PI3K-AKT signalling to inhibit the protein levels of FOXO1 in a mouse model of acute cardiac ischaemia-reperfusion [75]. In summary, we believe that when acute stress occurs, EPI or NE acts on β_2_-AR, which plays a protective role on the body; however, when the body is unable to cope with stress, we believe that FOXO1 will have sustained activation.

In addition, we found that the p-NF-κB p65 level was significantly reduced after the addition of AS1842865, which was the blocking agent of FOXO1 (Figure 8). There is little indirect evidence for the relationship between FOXO1 and NF-κB. It has been reported that the NF-κB pathway is activated by TNF-α and is significantly reduced by FOXO1 knockdown [76]. Other articles have suggested that, under the same condition, both FOXO1 and NF-κB tend to increase or decrease in parallel. For example, RELA (NF-κB p65) has a similar role to FOXO1, and both may prevent signal transducers and activators of transcription 3 (STAT3)-mediated leptin activation of a pro-opiomelanocortin (Pomc) promoter [77]. Patients with muscle atrophy from type 2 diabetes mellitus show increased expression of NF-κB and higher expression of the atrophy transcription factor FOXO1 in skeletal muscle [78]. Our study provides evidence that will assist in the study of the relationship between FOXO1 and NF-κB. In summary, we believe that FOXO1 may play a mediating role between β_2_-AR and NF-κB.

## 4. Materials and Methods

### 4.1. Animal Treatments

A total of 150 female ICR mice and 50 male mice (being used for mating, 8 weeks of age; Vital River Laboratory Animal Technology Co. Ltd., Beijing, China) were housed under conventional conditions (at a relative humidity of 50 ± 10% and a temperature of 21 ± 1 °C) with a regular 10 h dark/14 h light cycle (with lights on at 7:00 a.m.). First, the mice were maintained in a room for a week in order to adapt to the environment. Oestrus mice were placed with a sexually experienced male at night, and females with vaginal sperm plugs were then considered to be P1 (Pregnancy Day 1) on the next morning. Mice were divided into control (*n* = 50) and restraint stress groups (*n* = 50), each group were sacrificed on NP5 (Non-pregnancy, *n* = 10), P5 (*n* = 10), P10 (*n* = 10), P15 (*n* = 10), and P20 (*n* = 10). Restraint stress female mice were individually placed into transparent and ventilated plastic centrifuge tubes (they were able to breathe freely but were unable to escape) to limit their movements for 4 h (from 8:00 a.m. to 12:00 p.m.), and the female mice of the control group were freedom in the cage. Restraint stress and control conditions were initiated on NP1 (Non-Pregnancy Day 1, during the dioestrus period), P1 (Pregnancy Day 1), P6 (Pregnancy Day 6), P11 (Pregnancy day 11), and P16 (Pregnancy Day 16) until the mice were sacrificed on NP5 P5, P10, P15, and P20. The weight of the female mice was measured daily. The definition of body weight gain was the final weight minus the weight before stress. After the mouse was dissected, the liver, spleen, kidney, heart, ovary, uterus, and thymus were weighed. The organ index was expressed as the ratio of the weight of the organ to the body weight. 20 normal pregnancy mice (P5) were used to isolate ESCs to explore the signal pathway in vitro. Finally, 30 normal pregnant mice were divided into control group (*n* = 6), stress group (*n* = 8), control + AS group (*n* = 8), and stress+ AS group (*n* = 8) on P1. Stress+ AS group were treated by subcutaneous injection with FOXO1 inhibitor (AS1842856 (AS), 25 mg/kg at 6 p.m. every day until P5) and restraint stress. All animal procedures were approved by the China Agricultural University Institutional Animal Care and Use Committee (AW18079102-3-2).

### 4.2. Plasma and Tissue Preparations

Females mice were anaesthetized with 2% pentobarbital (4 mL/kg body weight, i.p.) on NP5, P5, P10, P15, and P20 immediately following the treatment. Plasma samples with eyeball extraction were collected to detect CORT, NE, and E2. Some of the uterine specimens were rapidly dissected, excluding the embryos, and stored in liquid nitrogen for protein analysis and mRNA analysis. Some of the uterine was immediately fixed in 4% paraformaldehyde for 48 h.

### 4.3. Quantitative Real-Time Polymerase Chain Reaction

Total RNA was extracted using TRIzol reagent (CW0580A, CoWin Biotech Co., Inc., Beijing, China). The concentration and purity of RNA was measured with a NanoPhotometer (P330, Implen, Munich, Germany). The concentration of RNA was 1500–1800 ng/μL. Afterward, 2 μg of total RNA were mixed with reverse transcriptase, and other reagents from the cDNA were synthesized using the GoScript^TM^ Reverse Transcription System (A5001, Promega, Madison, WI., USA). The cDNA was diluted 10 times to conduct polymerase chain reactions. Primers were synthesized by Invitrogen Trading of Shanghai. We used the following primers: FOXO1 primer (forward, 5′-GTGAAGAGCGTGCCCTACT-3′; reverse, 5′-GATTGAGCATCCACCAAGAAC-3′); FOXO3 primer (forward, 5′-CTCTCAGGCTCCTCACTGTA-3′; reverse, 5′-ATGAGTTCACTACGGATGAT-3′); FOXO4 primer (forward, 5′-TCATCAAGGTTCACAACGAGGC-3′; reverse, 5′-AGGACAGACGGCTTCTTCTTGG-3′); β_2_-AR primer (forward, 5′-TCACTCAGGAACGGGACGAAG-3′; reverse, 5′-CAGCACACGCCAAGGAGATTATG-3′); IL-2 primer (forward, 5′-TGAACTTGGACCTCTGCG -3′; reverse, 5′-AGGGCTTGTTGAGATGATGC-3′); IL-6 primer (forward, 5′- CTGCAAGAGACTTCCATCCAG-3′; reverse, 5′-AGTGGTATAGACAGGTCTGTTGG-3′); TNF-α primer (forward, 5′-CAGGCGGTGCCTATGTCTC-3′; reverse, 5′-CGATCACCCCGAAGTTCAGTAG-3′); β-actin primer (forward, 5′-TGCTGTCCCTGTATGCCTCTG-3′; reverse, 5′-TTGATGTCACGCACGATTTCC-3′). β-actin was used as the housekeeping gene. qRT-PCR amplification was performed by Roche LightCycler480 (Roche, Basel, Switzerland). Primers of qRT-PCR efficiency with one specific melting peak were used for the analysis. In this experiment, we used a 20 μL system containing 0.4 μL of forward primers, 0.4 μL of reverse primers, a 10 μL SYBR Green qPCR Master Mix kit (without ROX) (Q121-02, Vazyme Biotech Co., Ltd., Nanjing, China), 7.2 μL of nuclease free water, and 2 μL of cDNA. Relative target gene expression was obtained by normalizing the result to β-actin. The calculation method of mRNA expression was 2^−△Ct^. The value of NP5 was regarded as 1 and was used as the base level for assessment. Each sample was tested in triplicate.

### 4.4. Measurement of Plasma CORT, NE, Blood Glucose, and E2 Concentration

Total plasma CORT and NE concentrations were measured using a competitive enzyme-linked immunosorbent assay, the Mouse CORT enzyme linked immunosorbent assay (ELISA) Kit (LBTR-EL-1490, Beijing Limbo Terry Technology Development Co., Ltd., Beijing, China) and Mouse NE ELISA Kit (LBTR-EL-1487, Beijing Limbo Terry Technology Development Co., Ltd., Beijing, China). All of the tests were performed according to the manufacturers’ instructions. The antigen and antibodies were combined with biotinylated secondary antibodies and bound with streptavidin-horseradish peroxidase (HRP). The immune complex was formed and incubated at 37 °C for 60 min. After the colour was developed at 37 °C, the OD value was measured with a microplate reader, and the NE and CORT contents of the sample were calculated according to the standard curve (NE: y = 274.79x−22.276, R^2^ = 0.9908; CORT: y = 34.542x−3.782, R^2^ = 0.9886). The limit of detection for the NE assay ranged from 5 to 1000 ng/L. The limit of detection for the CORT assay ranged from 0.5 to 100 ng/mL. Five plasma samples were included in each group. Each sample was tested in triplicate.

E2 was detected via radioimmunoassay (RIA). Radioimmunoassay is a labelled immunoassay that uses radionuclides as a marker for the quantitative determination of antigens in a sample to be tested. The labelled antigen (Ag*) and the non-labelled antigen (Ag) compete with the specific antibody (Ab) for the binding reaction. The fitting was performed using a logarithmic measure-log pair logarithm (log(dose)~logit (Bi/B0)) mathematical model or a four-parameter mathematical model. Afterward, the percent binding rate of each tube was calculated: S0 (number of cpm) was B0, each calibration tube Si (number of cpm) was Bi, and the percent binding ratio of each tube was obtained. Using logX ~ logitY regression, the formula was obtained (logitY = 3.3388–0.6342logX). The content of the sample can be obtained by the formula. Five plasma samples were included in each group. Each sample was tested in triplicate.

Blood glucose was measured with a blood glucose meter (GC14906201, ACCU-CHEK, Basel, Switzerland, Germany), and the blood was collected through the tail vein. Five blood samples were included in each group. Each sample was tested in triplicate.

### 4.5. Measurements of Oxidative Stress-Related Enzymes

Immediately after the mice were sacrificed, the uterus was rapidly dissected, and the embryos were removed, frozen in liquid nitrogen, and stored. The uterus with removed embryos was weighed and placed in a 0.9% saline solution (1:9) to prepare a uterine homogenate. Finally, supernatants were extracted by centrifugation (2000× *g*, 20 min). The total protein concentration was measured using a BCA Protein Assay Kit (CW0014S, CoWin Biotech Co., Inc., Beijing, China). Total antioxidant capacity (T-AOC) (A015), SOD (A001-1), GSH-Px (A005), and MDA (A003-1) assay kits were purchased from Nanjing Jiancheng Bioengineering Institute (Nanjing, China). GSH-Px and SOD are well-known scavenger enzymes that protect cells from oxidative stress. SOD was detected by the xanthine oxidase method, and GSH-Px was determined by the rate at which it was converted to the enzymatic reaction of oxidized glutathione disulfide (GSSG). T-AOC was detected by converting Fe^3+^ to Fe^2+^. Those values were expressed as U/mg of protein. MDA is responsible for inducing oxidative stress, and it reacts with thiobarbituric acid to form a red complex and is expressed as nmol/mg of protein. These results were detected at specific wavelengths (T-SOD: 550 nm, GSH-Px: 412 nm, T-AOC: 520 nm, and MDA: 532 nm). Five tissue samples were included in each group, and each sample was tested in triplicate.

### 4.6. Western Blotting

Whole-tissue lysates were prepared from tissue, and protein concentrations were measured using a BCA protein assay reagent (CW0014S, CoWin Biotech Co., Inc., Beijing, China). These proteins were separated by 10% sodium dodecyl sulfate polyacrylamide gel electrophoresis (SDS-PAGE), followed by electrotransfer onto a polyvinylidene fluoride membrane (Millipore, Billerica, MA, USA). The membrane was blocked with 5% skim milk/0.5% Tween-20 in TBST for 2 h at room temperature. The primary antibody was probed with an anti-β-actin antibody (1:4000; CW0096M, CoWin Biotech Co., Inc., Beijing, China), an anti-FOXO1 antibody (1:1000, 2880T, Cell Signalling Technology, Beverly, MA, USA), an anti-RELA antibody (1:1000, total-NF-κB p65 (t-NF-κB p65), A1115, ABclonal Biotech Co., Ltd., Cambridge, MA, USA), an anti-phospho-RELA-S536 antibody (1:500, p-NF-κB p65, AP0475, ABclonal Biotech Co., Ltd., Cambridge, MA, USA), and an anti-ADRB2 antibody (1:1000, β_2_-AR, A1295, ABclonal Biotech Co., Ltd., Cambridge, MA, USA) overnight at 4 °C. Afterward, the membranes were washed in TBST, followed by a horseradish peroxidase-conjugated goat anti-mouse IgG (1:6000; CW0102M; CoWin Biotech Co., Inc., Beijing, China) for β-actin or goat anti-rabbit IgG (1:6000; CW0103M; CoWin Biotech Co., Inc., Beijing, China) for FOXO1, β_2_-AR, p-NF-κB p65, and t-NF-κB p65 for 2 h at room temperature. Immunoblotting was visualized with an ECL Western Blot Kit (CW0049M, CoWin Biotech Co., Inc., Beijing, China). The bands obtained in the blots were scanned and analysed with ImageJ (National Institutes of Health, Bethesda, MD, USA). The data are expressed as the integral optical density (IOD) of the bands, normalized to the IOD of the corresponding β-actin bands. Five tissue samples were included in each group. Each sample was repeated three times.

### 4.7. Immunohistochemistry

FOXO1 was localized by immunohistochemistry. The uterus taken at P5 was processed into paraffin sections. All sections (5 μm) were routinely dewaxed in xylene and rehydrated with graded concentrations of ethanol. Afterward, all slides were subjected to antigen retrieval by 0.01 M sodium citrate hydrochloric acid buffer. Tissue sections were washed 3 times with phosphate-buffered saline (PBS, pH 7.0) for 5 min each. Endogenous peroxidase activity was blocked with 3% H_2_O_2_ for 30 min. Non-specific staining was blocked with 5% normal goat serum for 30 min. The sections were incubated at 4 °C overnight with anti-FOXO1 antibody (1:100, 2880T, Cell Signalling Technology, Beverly, MA, USA). Next, the slides were washed with PBS and incubated sequentially with 1:400 biotin-conjugated goat anti-rabbit IgG (Beijing CoWin Biotech Co., Ltd., Beijing, China) for 2 h. The slides were washed with PBS and incubated sequentially with 1:400 HRP-streptavidin (Beijing CoWin Biotech Co., Ltd., Beijing, China) for 2 h. The immunoreactivity was revealed by diaminobenzidine (DAB) treatment, and nuclei were counterstained with haematoxylin for 5 min. The sections incubated with PBS instead of primary antibody served as negative controls. Images were acquired using an upright DP72 microscope (Olympus, Tokyo, Japan).

### 4.8. Cell Culture with Drug Treatment

ESCs taken during mouse pregnancy (P5) were cultured according to a reported method [79]. The uterus was placed in Hanks balanced saline solution (HBSS) containing 1% penicillin–streptomycin to clean the uterus. Afterward, the uterus was digested in a mixed digestive solution containing 2.5% pancreatin and 0.25% trypsin for 1 h in a 4 °C shaker; afterward, the uterus was left to stand at 25 °C for 45 min and at 37 °C for 15 min. The uterus was then moved and placed in DMEM medium containing 10% foetal bovine serum for 5 min and then placed in cold HBSS and vortexed for 10 s; this step was repeated twice. The uterus was placed in a mixed digestive solution containing 0.05% trypsin-EDTA and 0.1% collagenase I and shaken at 37 °C for 30 min. The uterus was washed in cold HBBS, and the uterus was placed into a DMEM/F12 medium containing 10% foetal bovine serum and vortexed for 10 s. The cells collected with DMEM/F12 complete medium were passed through a 40 μm sieve to collect single cells with 500 g centrifugation for 7 min. Resuspended cells were cultured in a 12-well plate at 1 × 10^6^ cells/well. The cells were cultivated in a 37 °C, 5% CO_2_ incubator for 45 min; afterward, the non-adherent cells were discarded, and the DMEM/F12 complete medium was added to the cells to cultivate for 24 h. Finally, the cells were starved for 6 h with DMEM/F12 basic medium.

Addition of drugs: Formoterol hemifumarate (For) (10^−8^ M, 1448, Tocris Bioscience, Minneapolis, MN, USA) is a selective agonist of β_2_-AR. Butoxamine hydrochloride (Butox) (10^−6^ M, B1385, Sigma, St. Louis, MO, USA) is the blocking agent of β_2_-AR, but its specific mechanism of action is not yet clear. AS1842865 (8 × 10^−7^ M, HY-100596, Med Chem Express, Brea, CA, USA) is an inhibitor of FOXO1. Pyrrolidine dithiocarbamate ammonium (PDTC) (10^−6^ M, 5108-96-3, Tocris Bioscience, Minneapolis, MN, USA) [80] is an inhibitor of NF-κB, as it prevents an increase in NOS mRNA. The cells were cultured with different drugs and named Cell+For (F), Cell+For+Butox (F+B), Cell+For+AS1842865 (F+A), Cell+For+PDTC (F+P), Cell+For+AS1842865+PDTC (F+A+P), Cell+For+Butox+AS1842865 (F+B+A), Cell+For+Butox+PDTC (F+B+P), Cell, and 0.02% DMSO. The inhibitor was added for 30 min, followed by the addition of For or not for 24 h [80]. Cells were collected with protein lysate and subjected to ultrasonic vibration. The supernatant was collected by centrifugation at 14,000 rpm for 10 min to detect the protein levels of FOXO1, β_2_-AR, and p NF-κB p65/t-NF-κB p65 in different groups. The same experiment was performed four times, and each sample was tested in triplicate.

### 4.9. Statistical Methods

The data were analysed with SPSS 18.0 (SPSS Inc., Chicago, IL, USA). To analyse the effects of restraint stress and different gestational stages on the experimental data, a two-way analysis of variance was performed. A one-way analysis of variance (ANOVA) was performed, followed by Duncan’s post hoc test, to analyse the differences of all indexes at different age in the control and stress group. Different uppercase letters (A, B, C, D, and E) represent the differences between NP5, P5, P10, P15 and P20 in the stressed group, and different lowercase letters (a, b, c, d, and e) represent the differences between NP5, P5, P10, P15 and P20 in the control group. Treatment groups are significantly different from each other if no labelling letter (a to e or A to E) is shared between groups. The significant difference between the control and stress mice at the same gestational stage were evaluated by an independent sample *t* test (two-tailed test). The data are expressed as the mean ± standard error of mean (SEM). In this result, the percentage ((Value _Stress_-Value _Control_)/Value _Control_) represented that the ratio of increase or decrease in stress group compared with control group in vivo. * *p* < 0.05 was used to indicate that the difference was significant, and # *p* < 0.01 indicated that the difference was extremely significant compared with the corresponding control group. In vitro, the stimulate index of ESCs (see Figures S2 and S3 of Supplement Material 1) was calculated as follows:

SI = OD_570_ treatment group/OD_570_ control group

where * represents the difference compared with the For group; + represents the difference compared with the For+Butox group. $ represents the difference compared with the For+Butox+As group. A single symbol indicates that the difference was significant, and a double symbol indicates that the difference was extremely significant, compared with the corresponding groups.

## 5. Conclusions

**Figure 9:**
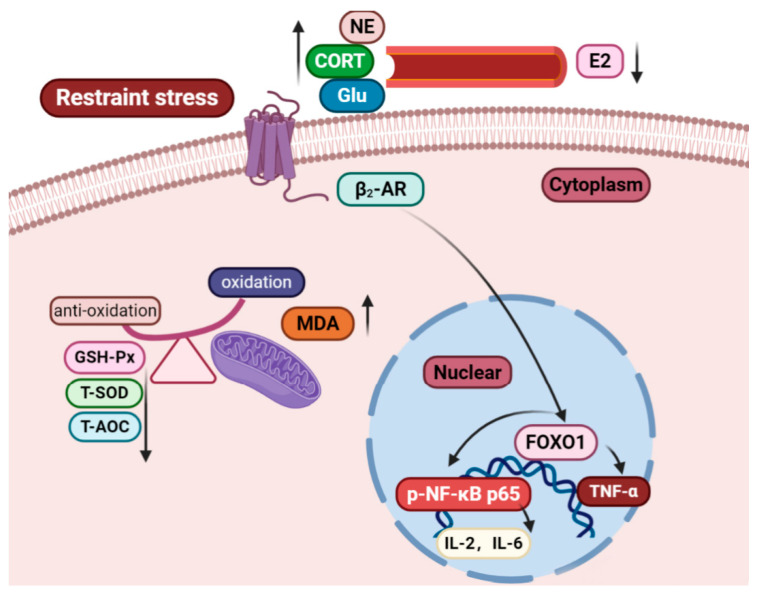
The antioxidative levels and relative protein levels and the mediating role of FOXO1 in p-NF-κB p65 and β_2_-AR. Restraint stress caused disorder in the endocrine system, leading to a significant increase in levels of the oxidative stress-related factors FOXO and β_2_-AR and suggesting a cascade of oxidative stress in pregnant mothers under oxidative stress. β_2_-AR affected the activity of FOXO1 while inhibiting the activity of p-NF-κB p65 and then inhibited the target gene of p-NF-κB p65. FOXO1 may play an important mediating role between β_2_-AR and NF-κB p65. The upward arrow represents an increase, while the downward arrow represents a decrease.

In vivo, restraint stress triggered the generation of oxidative stress, resulting in a state of imbalance between levels of oxidative and antioxidative stress, and leading to the loss of body weight in pregnant mothers. Restraint stress effected the index of organs and promoted an increase in CORT, NE, and blood glucose levels and a decrease in E2, leading to neuroendocrine disorders. Furthermore, β_2_-AR and FOXO in the uterus and ovary were activated. In vitro, FOXO1 may be a critical mediator in the crosstalk between β_2_-AR and p-NF-κB p65 in ESCs (Figure 9). This finding also provides evidence to support the study of human pregnancy-related diseases.

## Data Availability

The data used to support the findings of this study are available from the corresponding author upon request.

## Supplementary Materials

The following are available online at https://www.mdpi.com/1422-0067/22/3/1478/s1, **Figure S1:** Images of the liver, spleen, thymus, heart, kidney, ovary and uterus; **Figure S2:** The stimulation index of uterus ESCs after adding For; **Figure S3:** The stimulation index of uterus ESCs after adding AS1842865 before adding For; **Table S1:** Data of Section 2.1; **Table S2:** Data of Section 2.2; **Table S3:** Data of Section 2.3; **Table S4:** Data of Section 2.4; **Table S5:** Data of Section 2.5; **Table S6:** Data of Section 2.6; **Table S7:** Data of Section 2.8.

## Abbreviations

BWG	Body weight gain
Butox	Butoxamine hydrochloride
cAMP	Cyclic adenosine monophosphate
CORT	Corticosterone
CRH	Corticotropin-releasing hormone
HBSS	D’Hanks balanced saline solution
DAB	Diaminobenzidine
ESCs	Endometrial stromal cells
EPI	Epinephrine
FOXO	Forkhead transcription factor class O
For	Formoterol hemifumarate
GSH-Px	Glutathione peroxidase
GnRH	Gonadotropin releasing hormone
HRP	Horseradish peroxidase
HPA	Hypothalamic-pituitary-adrenal
HPG	Hypothalamic-pituitary-gonadal
IκB	Inhibitor of NF-κB
InsRes	Insulin resistance
IOD	Integral optical density
IL-18	Interleukin 18
IL-6	Interleukin-6
IL-8	Interleukin 8
IL-1β	Interleukin -1 beta
IUGR	Intrauterine growth restriction
ISO	Isoproterenol
MDA	Malondialdehyde
NE	Norepinephrin
NP1	Non-pregnancy Day 1
NP5	Non-pregnancy Day 5
NF-κB	Nuclear factor-kappa B
E2	Oestradiol
ANOVA	One-way analysis of variance
GSSG	Oxidized glutathione disulphide
P1	Pregnancy Day 1
P10	Pregnancy Day 10
P15	Pregnancy Day 15
P20	Pregnancy Day 20
P11	Pregnancy Day 11
P16	Pregnancy Day 16
P5	Pregnancy Day 5
P6	Pregnancy Day 6
PI3K-AKT	Phosphatidylinositol 3-kinase-serine/threonine kinase
Pomc	Pro-opiomelanocortin
PDTC	Pyrrolidine dithiocarbamate ammonium
qRT-PCR	Quantitative real-time polymerase chain reaction
ROS	Reactive oxygen species
RT-PCR	Reverse transcription-polymerase chain reaction
STAT3	Signal transducers and activators of transcription 3
siRNA	Small interfering RNA
SAS	Sympathetic-adrenal medullary system
SDS-PAGE	Sodium dodecyl sulfate polyacrylamide gel electrophoresis
SEM	Standard error of mean
TNF-α	Tumor necrosis factor-alpha
T-AOC	Total antioxidant capacity
T-SOD	Total superoxide dismutase
uNK	Uterus natural killer cell
β_2_-AR	β_2_-adrenergic receptor
