# Supplementary material for: The Role of the FOXO1/β2-AR/p-NF-κB p65 Pathway in the Development of Endometrial Stromal Cells in Pregnant Mice under Restraint Stress
Source: Int J Mol Sci. 2021 Feb 2;22(3):1478. doi: 10.3390/ijms22031478 (PMC7867244; doi:10.3390/ijms22031478)
Supplement: Supplementary file 1 [file ijms-22-01478-s001.zip › ijms-1049152-supplementary final.pdf]

International Journal of *Molecular Sciences*

MDPI

*Supplementary Material*

# The role of the FOXO1/$\beta_2$-AR/p-NF-κB p65 pathway in the development of endometrial stromal cells in pregnant mice under restraint stress

**Jiayin Lu [1], Yaoxing Chen [1], Zixu Wang [1], Jing Cao [1] and Yulan Dong [1,2,*]**

1. Laboratory of Neurobiology, College of Veterinary Medicine, China Agricultural University, Beijing Haidian 100193, China; lujiayin0324@cau.edu.cn (J.L.); yxchen@cau.edu.cn (Y.C.); zxwang@cau.edu.cn (Z.W.); caojing@cau.edu.cn (J.C.)
2. Key Laboratory of Precision Nutrition and Food Quality, Ministry of Education, China Agricultural University, Beijing Haidian 100193, China

* Correspondence: ylbcdong@cau.edu.cn; Tel./Fax: 010-62733013

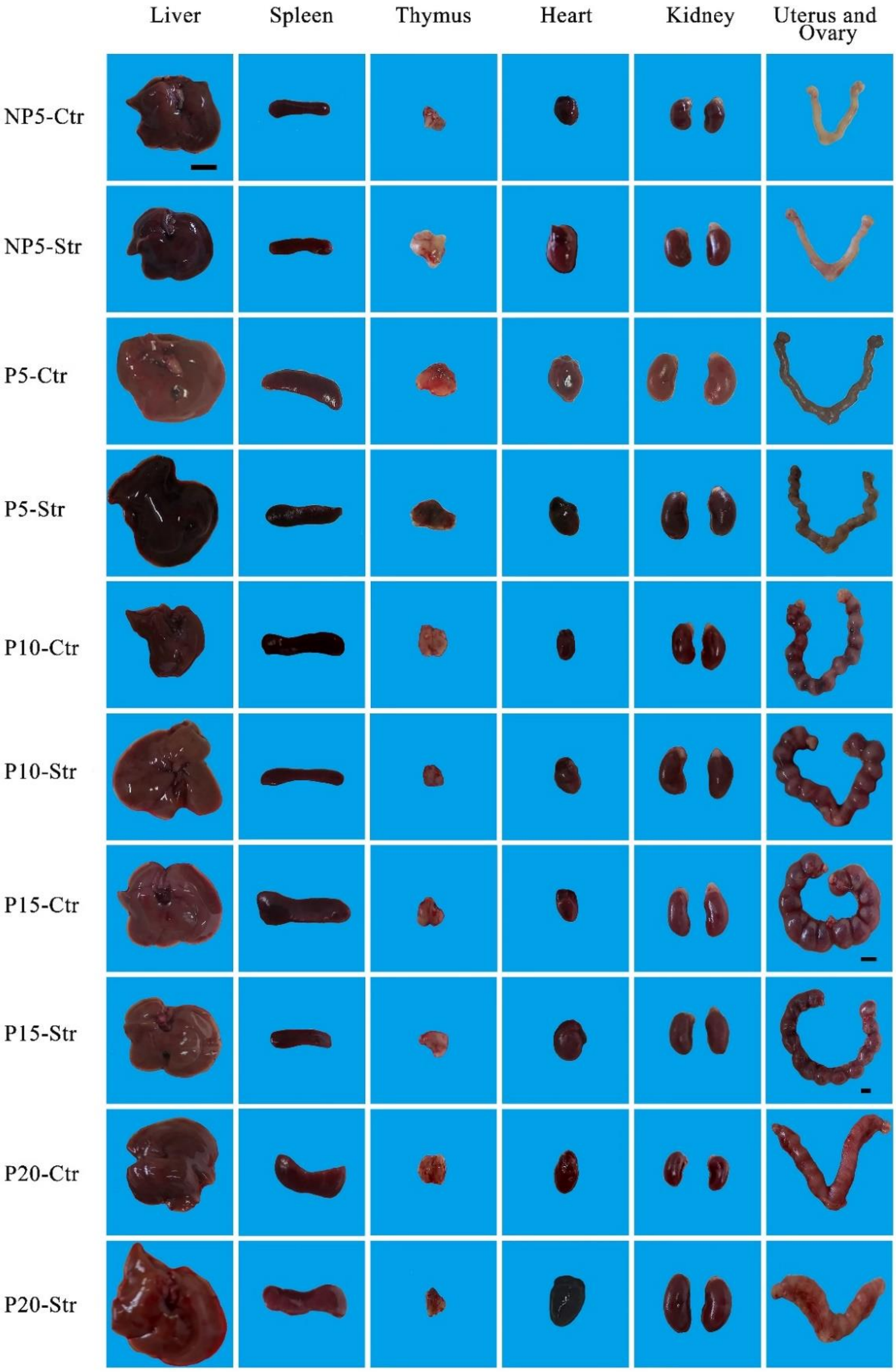


**Figure 1.** Images of the liver, spleen, thymus, heart, kidney, ovary and uterus. The morphology of the organ showed that restraint stress did not cause pathological damage to the organs in the pregnant mice. The scales of all pictures are based on the scale of the liver picture at non-pregnancy day 5 (NP5)-Ctr except for the uterus picture at pregnancy day 15 (P15)-Ctr and P15-Str. The scales of P15-Ctr and P15-Str are shown separately in the images. The scale bar is 0.5 cm. NP: non-pregnancy, P: pregnancy, Ctr: Control, Str: Stress.

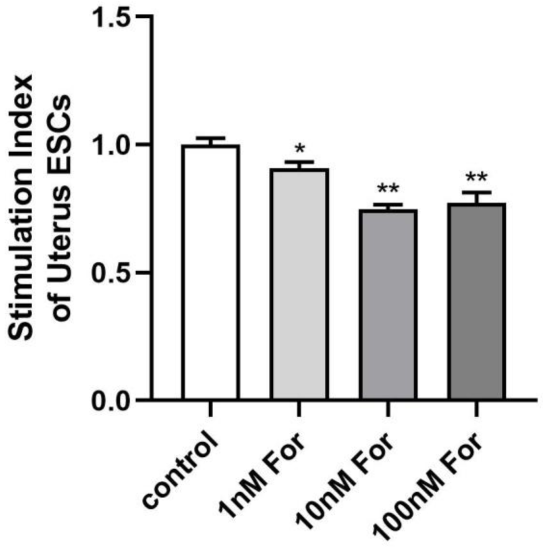


**Figure 2.** The stimulation index of uterus ESCs after adding For. Formoterol hemifumarate (For) is a selective agonist of β2-AR. The concentrations of For were set as 1 nM, 10 nM and 100 nM. In order to have a better effect and avoid the toxicity of the drug to the cells, we chose 10 nM as the final concentration. The stimulate index (SI) of uterus ESCs was calculated as follow: SI=$OD_{570}$ treatment group/ $OD_{570}$ control group; *$p < 0.05$ represent the significance compared with control and **$p < 0.01$ represent the extremely significance compared with control.

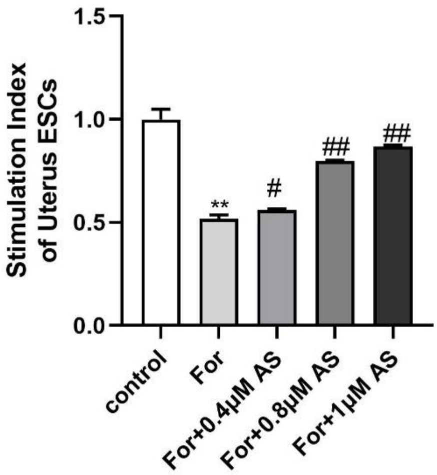


**Figure 3.** The stimulation index of uterus ESCs after adding AS1842865 before adding For. AS1842865 (AS) is an inhibitor of FOXO1. we followed our previous method of exploring inhibitor concentration (refer to the reference 79 in the manuscript R2). The concentrations of AS were set as 0.4 μM, 0.8 μM and 1 μM [1,2]. In order to have a better effect and avoid the toxicity of the drug to the cells, we chose 0.8μM as the final concentration. The stimulate index (SI) of uterus ESCs was calculated as follow: SI=$OD_{570}$ treatment group/ $OD_{570}$ control group

**$p < 0.01$ represent the significance compared with control. #$p < 0.05$ represent the significance compared with For group and ##$p < 0.01$ represent the extremely significance compared with For group.

[1] Zhang, Y.; Sun, C.; Xiao, G.; Shan, H.; Tang, L.; Yi, Y.; Yu, W.; Gu, Y. S-nitrosylation of the Peroxiredoxin-2 promotes S-nitrosoglutathione-mediated lung cancer cells apoptosis via AMPK-SIRT1 pathway. *Cell Death Dis*. 2019, 10, 329.

[2] Huang, W.; Cheng, C.; Shan, W.S.; Ding, Z.F.; Liu, F.E.; Lu, W.; He, W.; Xu, J.G.; Yin, Z.S. Knockdown of SGK1 alleviates the IL-1β-induced chondrocyte anabolic and catabolic imbalance by activating FoxO1-mediated autophagy in human chondrocytes. *FEBS J.* 2020, 287, 94-107.

**Table 1.** Data of Section 2.1.

| | Body Weight Gain (g) | | | *Statistical Value* | |
|---|---|---|---|---|---|
| **Days** | **Stress** | **Control** | **S-C** | **(S-C)/C%** | ***p*** |
| NP5 | -3.380 | 1.867 | -5.247 | -281.07% | < 0.001 |
| P5 | -2.229 | 0.533 | -2.762 | -517.86% | < 0.001 |
| P10 | -1.767 | 2.133 | -3.9 | -182.81% | < 0.001 |
| P15 | 4.867 | 8.650 | -3.783 | -43.74% | 0.004 |
| P20 | -13.800 | -16.350 | 2.55 | -15.60% | 0.006 |

Note: A positive number represents an increase, and a negative number represents a decrease.

**Table 2.** Data of Section 2.2.

| | **Index of Liver** | | | *Statistical Value* | | | **Index of Spleen** | | | *Statistical Value* | |
|---|---|---|---|---|---|---|---|---|---|---|---|
| **Day** | **Stress** | **Control** | **S-C** | **(S-C)/C%** | ***p*** | **Day** | **Stress** | **Control** | **S-C** | **(S-C)/C%** | ***p*** |
| NP5 | 4.90697 | 5.42912 | -0.52215 | **-9.62%** | 0.013 | NP5 | 0.40889 | 0.43008 | -0.02119 | **-4.93%** | 0.464 |
| P5 | 4.89944 | 5.83975 | -0.94031 | **-16.10%** | 0.008 | P5 | 0.39694 | 0.54179 | -0.14485 | **-26.74%** | 0.008 |
| P10 | 5.24635 | 6.20872 | -0.96237 | **-15.50%** | 0.059 | P10 | 0.57158 | 0.73079 | -0.15921 | **-21.79%** | 0.034 |
| P15 | 5.41683 | 5.91391 | -0.49708 | **-8.41%** | 0.010 | P15 | 0.41242 | 0.6138 | -0.20138 | **-32.81%** | 0.001 |
| P20 | 6.43489 | 6.94517 | -0.51028 | **-7.35%** | 0.048 | P20 | 0.3454 | 0.40189 | -0.05649 | **-14.06%** | 0.045 |
| | **Index of Thymus** | | | *Statistical Value* | | | **Index of Heart** | | | *Statistical Value* | |
| **Day** | **Stress** | **Control** | **S-C** | **(S-C)/C%** | ***p*** | **Day** | **Stress** | **Control** | **S-C** | **(S-C)/C%** | ***p*** |
| NP5 | 0.15298 | 0.25868 | -0.1057 | **-40.86%** | <0.001 | NP5 | 0.80162 | 0.76379 | 0.03783 | **4.95%** | 0.47 |
| P5 | 0.14454 | 0.2685 | -0.12396 | **-46.17%** | <0.001 | P5 | 0.68954 | 0.66445 | 0.02509 | **3.78%** | 0.754 |
| P10 | 0.12286 | 0.26503 | -0.14217 | **-53.64%** | <0.001 | P10 | 0.7015 | 0.67153 | 0.02997 | **4.46%** | 0.67 |
| P15 | 0.10587 | 0.18189 | -0.07602 | **-41.79%** | 0.005 | P15 | 0.71373 | 0.49308 | 0.22065 | **44.75%** | 0.003 |
| P20 | 0.08252 | 0.10266 | -0.02014 | **-19.62%** | 0.023 | P20 | 0.74319 | 0.60051 | 0.14268 | **23.76%** | 0.008 |
| | **Index of Kidney** | | | *Statistical Value* | | | **Index of Ovary** | | | *Statistical Value* | |
| **Day** | **Stress** | **Control** | **S-C** | **(S-C)/C%** | ***p*** | **Day** | **Stress** | **Control** | **S-C** | ***(S-C)/C%*** | ***p*** |
| NP5 | 1.34465 | 1.2548 | 0.08985 | **7.16%** | 0.228 | NP5 | 0.08831 | 0.11913 | -0.03082 | **-25.87%** | 0.008 |
| P5 | 1.34318 | 1.26207 | 0.08111 | **6.43%** | 0.242 | P5 | 0.08656 | 0.08995 | -0.00339 | **-3.77%** | 0.742 |
| P10 | 1.46352 | 1.35687 | 0.10665 | **7.86%** | 0.109 | P10 | 0.08761 | 0.10492 | -0.01731 | **-16.50%** | 0.016 |
| P15 | 1.11526 | 0.89607 | 0.21919 | **24.46%** | 0.019 | P15 | 0.07478 | 0.07781 | -0.00303 | **-3.89%** | 0.626 |
| P20 | 1.39173 | 1.19195 | 0.19978 | **16.76%** | 0.003 | P20 | 0.11079 | 0.11101 | -0.00022 | **-0.20%** | 0.982 |
| | **Index of Uterus** | | | *Statistical Value* | | | | | | | |
| **Day** | **Stress** | **Control** | **S-C** | ***(S-C)/C%*** | ***p*** | | | | | | |
| NP5 | 0.35355 | 0.46579 | -0.11224 | **-24.10%** | <0.001 | | | | | | |
| P5 | 0.48392 | 0.64108 | -0.15716 | **-24.51%** | 0.016 | | | | | | |
| P10 | 2.91252 | 3.65106 | -0.73854 | **-20.23%** | 0.076 | | | | | | |
| P15 | 20.56701 | 23.51922 | -2.95221 | **-12.55%** | 0.065 | | | | | | |
| P20 | 2.38273 | 3.00347 | -0.62074 | **-20.67%** | 0.047 | | | | | | |

Note: A positive number represents an increase, and a negative number represents a decrease.

**Table 3.** Data of Section 2.3.

| **Corticosterone (CORT) (ng/mL)** | | | | ***Statistical Value*** | | **Norepinephrine (NE) (pg/mL)** | | | | ***Statistical Value*** | |
|---|---|---|---|---|---|---|---|---|---|---|---|
| **Day** | **Stress** | **Control** | **S-C** | **(S-C)/C%** | ***p*** | **Day** | **Stress** | **Control** | **S-C** | **(S-C)/C%** | ***p*** |
| NP5 | 24.111705 | 18.063259 | 6.048446 | **33.48%** | 0.001 | NP5 | 102.1530071 | 45.3993925 | 56.75361 | **125.01%** | < 0.001 |
| P5 | 23.427535 | 19.88365025 | 3.543885 | **17.82%** | 0.044 | P5 | 123.8453881 | 95.6403073 | 28.20508 | **29.49%** | < 0.001 |
| P10 | 28.65555275 | 21.53749289 | 7.11806 | **33.05%** | 0.040 | P10 | 219.1187211 | 192.8681529 | 26.25057 | **13.61%** | < 0.001 |
| P15 | 21.44841079 | 19.03715933 | 2.411251 | **12.67%** | 0.010 | P15 | 187.2831897 | 155.8384863 | 31.4447 | **20.18%** | < 0.001 |
| P20 | 26.4557405 | 18.7864075 | 7.669333 | **40.82%** | < 0.001 | P20 | 211.8443813 | 168.5549137 | 43.28947 | **25.68%** | < 0.001 |
| **Blood Glucose (mmol/L)** | | | | ***Statistical Value*** | | **Estradiol (E2) (pg/mL)** | | | | ***Statistical Value*** | |
| **Day** | **Stress** | **Control** | **S-C** | **(S-C)/C%** | ***p*** | **Day** | **Stress** | **Control** | **S-C** | **(S-C)/C%** | ***p*** |
| NP5 | 8.533333333 | 6 | 2.533333 | **42.22%** | 0.007 | NP5 | 30.4163333 | 34.0308333 | -3.6145 | **-10.62%** | 0.131 |
| P5 | 8.675 | 6.8 | 1.875 | **27.57%** | < 0.001 | P5 | 13.8928333 | 27.5972500 | -13.7044 | **-49.66%** | < 0.001 |
| P10 | 8.333333333 | 6.633333333 | 1.7 | **25.63%** | 0.003 | P10 | 26.9551667 | 34.3950000 | -7.43983 | **-21.63%** | 0.004 |
| P15 | 7.9 | 7.075 | 0.825 | **11.66%** | 0.047 | P15 | 19.5103333 | 23.9883333 | -4.478 | **-18.67%** | 0.085 |
| P20 | 7.966666667 | 6.333333333 | 1.633333 | **25.79%** | 0.002 | P20 | 22.09 | 20.005667 | 2.084333 | **10.42%** | 0.143 |

Note: A positive number represents an increase, and a negative number represents a decrease.

**Table 4.** Data of Section 2.4.

| **T-SOD (U/mg protein)** | | | | ***Statistical Value*** | | **GSH-PX (U/mg protein)** | | | | ***Statistical Value*** | |
|---|---|---|---|---|---|---|---|---|---|---|---|
| **Day** | **Stress** | **Control** | **S-C** | **(S-C)/C%** | ***p*** | **Day** | **Stress** | **Control** | **S-C** | **(S-C)/C%** | ***p*** |
| NP5 | 3.423 | 3.471299 | -0.0483 | **-1.39%** | 0.776 | NP5 | 72.006737 | 68.69 | 3.316737 | **4.83%** | 0.589 |
| P5 | 2.873 | 3.499159 | -0.62616 | **-17.89%** | <0.001 | P5 | 129.293 | 182.702 | -53.409 | **-29.23%** | 0.001 |
| P10 | 2.618 | 3.04502 | -0.42702 | **-14.02%** | 0.011 | P10 | 108.76 | 131.94 | -23.18 | **-17.57%** | <0.001 |
| P15 | 2.647 | 2.53502 | 0.11198 | **4.42%** | 0.330 | P15 | 151.389 | 153.107 | -1.718 | **-1.12%** | 0.461 |
| P20 | 2.237 | 2.813368 | -0.57637 | **-20.49%** | <0.001 | P20 | 152.542 | 163.498 | -10.956 | **-6.70%** | <0.001 |
| **T-AOC (U/mg protein)** | | | | ***Statistical Value*** | | **MDA (nmol/mg protein)** | | | | ***Statistical Value*** | |
| **Day** | **Stress** | **Control** | **S-C** | **(S-C)/C%** | ***p*** | **Day** | **Stress** | **Control** | **S-C** | **(S-C)/C%** | ***p*** |
| NP5 | 0.762 | 1.497 | -0.735 | **-49.10%** | <0.001 | NP5 | 6.189224 | 6.017 | 0.172224 | **2.86%** | 0.265 |
| P5 | 0.45 | 0.744 | -0.294 | **-39.52%** | <0.001 | P5 | 4.63156 | 2.834 | 1.79756 | **63.43%** | <0.001 |
| P10 | 0.908 | 1.464 | -0.556 | **-37.98%** | <0.001 | P10 | 6.259932 | 2.912 | 3.347932 | **114.97%** | <0.001 |
| P15 | 0.998 | 1.245 | -0.247 | **-19.84%** | 0.001 | P15 | 3.521836 | 2.159 | 1.362836 | **63.12%** | <0.001 |
| P20 | 0.974 | 1.321 | -0.347 | **-26.27%** | 0.012 | P20 | 6.757903 | 1.657 | 5.100903 | **307.84%** | <0.001 |

Note: A positive number represents an increase, and a negative number represents a decrease.

**Table 5.** Data of Section 2.5.

| **FOXO1 mRNA of Ovary** | | | | ***Statistical Value*** | | **FOXO1 mRNA of Uterus** | | | | ***Statistical Value*** | |
|---|---|---|---|---|---|---|---|---|---|---|---|
| **Day** | **Stress** | **Control** | **S-C** | **(S-C)/C%** | ***p*** | **Day** | **Stress** | **Control** | **S-C** | **(S-C)/C%** | ***p*** |
| NP5 | 0.060791145 | 0.048486482 | 0.012305 | **25.38%** | < 0.001 | NP5 | 0.035851434 | 0.027789183 | 0.008062 | **29.01%** | < 0.001 |
| P5 | 0.142046154 | 0.098447619 | 0.043599 | **44.29%** | < 0.001 | P5 | 0.05943 | 0.022169444 | 0.037261 | **168.07%** | < 0.001 |
| P10 | 0.08182963 | 0.067015 | 0.014815 | **22.11%** | < 0.001 | P10 | 0.015600478 | 0.011467922 | 0.004133 | **36.04%** | < 0.001 |
| P15 | 0.064435 | 0.065276923 | -0.00084 | **-1.29%** | 0.796 | P15 | 0.014635714 | 0.01432 | 0.000316 | **2.20%** | 0.177 |
| P20 | 0.047628571 | 0.050434511 | -0.00281 | **-5.56%** | 0.192 | P20 | 0.020234783 | 0.016259262 | 0.003976 | **24.45%** | < 0.001 |
| **FOXO3 mRNA of Ovary** | | | | ***Statistical Value*** | | **FOXO3 mRNA of Uterus** | | | | ***Statistical Value*** | |
| **Day** | **Stress** | **Control** | **S-C** | **(S-C)/C%** | ***p*** | **Day** | **Stress** | **Control** | **S-C** | **(S-C)/C%** | ***p*** |
| NP5 | 0.0059517 | 0.004007133 | 0.001945 | **48.53%** | < 0.001 | NP5 | 0.005377738 | 0.003015491 | 0.002362 | **78.34%** | < 0.001 |
| P5 | 0.0045244 | 0.00294 | 0.001584 | **53.89%** | < 0.001 | P5 | 0.003146275 | 0.002187778 | 0.000958 | **43.81%** | 0.001 |
| P10 | 0.0049243 | 0.002835926 | 0.002088 | **73.64%** | < 0.001 | P10 | 0.001642614 | 0.001262734 | 0.00038 | **30.08%** | < 0.001 |
| P15 | 0.0032111 | 0.002508889 | 0.000702 | **27.99%** | < 0.001 | P15 | 0.000741571 | 0.000483045 | 0.000259 | **53.52%** | < 0.001 |
| P20 | 0.0079415 | 0.008387174 | -0.00045 | **-5.31%** | 0.357 | P20 | 0.002693 | 0.00234024 | 0.000353 | **15.07%** | 0.006 |
| **FOXO4 mRNA of Ovary** | | | | ***Statistical Value*** | | **FOXO4 mRNA of Uterus** | | | | ***Statistical Value*** | |
| **Day** | **Stress** | **Control** | **S-C** | **(S-C)/C%** | ***p*** | **Day** | **Stress** | **Control** | **S-C** | **(S-C)/C%** | ***p*** |
| NP5 | 0.00041308 | 0.000470085 | -0.000057005 | **-12.13%** | 0.002 | NP5 | 0.000927489 | 0.000611249 | 0.000316 | **51.74%** | < 0.001 |
| P5 | 0.00052805 | 0.000322632 | 0.000205418 | **63.67%** | < 0.001 | P5 | 0.000569067 | 0.000451727 | 0.000117 | **25.98%** | 0.016 |
| P10 | 0.000389211 | 0.00038 | 9.211E-06 | **2.42%** | 0.48 | P10 | 0.000209812 | 0.00020671 | 3.1E-06 | **1.50%** | 0.808 |
| P15 | 0.000338867 | 0.000235095 | 0.000103772 | **44.14%** | < 0.001 | P15 | 0.000206905 | 0.000149474 | 5.74E-05 | **38.42%** | < 0.001 |
| P20 | 0.000604421 | 0.000426351 | 0.00017807 | **41.77%** | < 0.001 | P20 | 0.000364889 | 0.000210806 | 0.000154 | **73.09%** | < 0.001 |
| **FOXO1 protein of Ovary** | | | | ***Statistical Value*** | | **FOXO1 protein of Uterus** | | | | ***Statistical Value*** | |
| **Day** | **Stress** | **Control** | **S-C** | **(S-C)/C%** | ***p*** | **Day** | **Stress** | **Control** | **S-C** | **(S-C)/C%** | ***p*** |
| NP5 | 0.63805084 | 0.57904057 | 0.05901 | **10.19%** | 0.441 | NP5 | 0.707232 | 0.232096 | 0.475136 | **204.72%** | 0.003 |
| P5 | 1.04394783 | 0.66675639 | 0.377191 | **56.57%** | 0.005 | P5 | 0.76552 | 0.544843 | 0.220677 | **40.50%** | < 0.001 |
| P10 | 0.79489277 | 0.52759203 | 0.267301 | **50.66%** | 0.001 | P10 | 0.979896 | 0.76106 | 0.218836 | **28.75%** | 0.011 |
| P15 | 0.7926329 | 0.50429267 | 0.28834 | **57.18%** | 0.010 | P15 | 0.906055 | 0.426266 | 0.479789 | **112.56%** | < 0.001 |
| P20 | 0.63740861 | 0.53859903 | 0.09881 | **18.35%** | 0.166 | P20 | 0.691471 | 0.536549 | 0.154922 | **28.87%** | 0.035 |

**Note:** A positive number represents an increase, and a negative number represents a decrease.

**Table 6.** Data of Section 2.6.

| **$\beta_2$-AR mRNA of Ovary** | | | | ***Statistical Value*** | | **$\beta_2$-AR mRNA of Uterus** | | | | ***Statistical Value*** | |
|---|---|---|---|---|---|---|---|---|---|---|---|
| **Day** | **Stress** | **Control** | **S-C** | **(S-C)/C%** | ***p*** | **Day** | **Stress** | **Control** | **S-C** | **(S-C)/C%** | ***p*** |
| NP5 | 0.007856879 | 0.004959122 | 0.002898 | **58.43%** | < 0.001 | NP5 | 0.007214122 | 0.006070479 | 0.001144 | **18.84%** | 0.002 |
| P5 | 0.004338519 | 0.002108932 | 0.00223 | **105.72%** | < 0.001 | P5 | 0.008513077 | 0.004739422 | 0.003774 | **79.62%** | < 0.001 |
| P10 | 0.003153518 | 0.001834521 | 0.001319 | **71.90%** | < 0.001 | P10 | 0.003351208 | 0.002909974 | 0.000441 | **15.16%** | 0.177 |
| P15 | 0.003032981 | 0.00261608 | 0.000417 | **15.94%** | 0.082 | P15 | 0.003116423 | 0.002561161 | 0.000555 | **21.68%** | 0.011 |
| P20 | 0.006805936 | 0.002627436 | 0.004179 | **159.03%** | < 0.001 | P20 | 0.003660538 | 0.002425531 | 0.001235 | **50.92%** | < 0.001 |
| **$\beta_2$-AR protein of Ovary** | | | | ***Statistical Value*** | | **$\beta_2$-AR protein of Uterus** | | | | ***Statistical Value*** | |
| **Day** | **Stress** | **Control** | **S-C** | **(S-C)/C%** | ***p*** | **Day** | **Stress** | **Control** | **S-C** | **(S-C)/C%** | ***p*** |
| NP5 | 0.86498913 | 0.50297686 | 0.362012269 | 71.97% | 0.002 | NP5 | 0.80790172 | 0.49909648 | 0.308805 | **61.87%** | 0.007 |
| P5 | 1.0503356 | 0.81466723 | 0.235668373 | 28.93% | 0.006 | P5 | 1.52109353 | 0.70840361 | 0.81269 | **114.72%** | 0.005 |
| P10 | 0.74334867 | 0.65393414 | 0.089414524 | 13.67% | 0.267 | P10 | 0.86952794 | 0.80389347 | 0.065634 | **8.16%** | 0.454 |
| P15 | 0.96346647 | 0.25860105 | 0.704865418 | 272.57% | < 0.001 | P15 | 0.58956191 | 0.5191016 | 0.07046 | **13.57%** | 0.276 |
| P20 | 0.86195584 | 0.86817985 | -0.006224008 | -0.72% | 0.949 | P20 | 0.51362147 | 0.3881846 | 0.125437 | **32.31%** | 0.031 |

Note: A positive number represents an increase, and a negative number represents a decrease.

**Table 7.** Data of Section 2.8.

| **Sequence** | | **p-NF-κB p65/t-NF-κB p65** | | | **FOXO1** | | | | | | **$\beta_2$-AR** | | |
|---|---|---|---|---|---|---|---|---|---|---|---|---|---|
| | **Group** | **Value** | **Statistic** | | **Value** | **Statistic** | | **Value** | **Statistic** | | **Value** | **Statistic** | |
| | | | **percent** | ***p*** | | **percent** | ***p*** | | **percent** | ***p*** | | **percent** | ***p*** |
| 1 | Control | 0.293468126 | - | - | 0.668034677 | - | - | | | | 0.180270179 | - | - |
| 2 | DMSO | 0.270392888 | - | - | 0.781934605 | - | - | | | | 0.249707915 | - | - |
| 3 | F+A+P | 0.312924618 | - | - | 0.989507911 | | - | | | | 0.349166209 | - | - |
| 4 | F+B+A | 0.3168023 | - | - | 0.72623707 | - | - | | | | 0.311256031 | - | - |
| 5 | F+B+P | 0.349263467 | - | - | 0.619001708 | - | - | | | | 0.373814589 | - | - |
| 6 | F+A | 0.299590907 | - | - | 0.969998089 | - | - | | | | 0.329948065 | - | - |
| 7 | F+P | 0.209154447 | - | - | 1.044371454 | - | - | | | | 0.384313817 | - | - |
| 8 | F+B | 0.464319652 | (F+B-F+B+A)/F+B 47.53% | 0.022 | 0.755693937 | (F-F+B)/F 33.14% | 0.001 | | | | 0.373068648 | - | - |
| 9 | F | 0.287261182 | (F+B-F)/F 61.64% | 0.009 | 1.13027014 | (F-C)/C 69.19% | 0.001 | | | | 0.340371475 | (F-C)/C 88.81% | 0.010 |
| | | **IL-2** | | | **IL-6** | | | **TNF-alpha** | | | **p-NF-κB p65/t-NF-κB p65** | | |
| 1 | Control | 0.000457 | - | - | 0.00055 | - | - | 0.000528 | - | - | 0.208074 | - | - |
| 2 | Stress | 0.000662 | (S-C)/C 44.94% | 0.021 | 0.001525 | (S-C)/C 177.14% | 0.001 | 0.002231 | (S-C)/C 322.70 | <0.001 | 0.415671 | (S-C)/C 99.77% | < 0.001 |
| 3 | Control+AS | 0.000453 | - | - | 0.000416 | - | - | 0.000702 | - | - | 0.225679 | - | - |
| 4 | Stress+AS | 0.000462 | (S-S+A)/S 30.25% | 0.032 | 0.000672 | (S-S+A)/S 55.95% | 0.001 | 0.000601 | (S-S+A)/S 73.08% | < 0.001 | 0.249011 | (S-S+A)/S 40.09 | < 0.001 |

Note: A positive number represents an increase, and a negative number represents a decrease.
